# Psychosocial Effects of Receiving Genome-Wide Polygenic Risk Information Concerning Type 2 Diabetes and Coronary Heart Disease: A Randomized Controlled Trial

**DOI:** 10.3389/fgene.2022.881349

**Published:** 2022-05-30

**Authors:** Otto Halmesvaara, Marleena Vornanen, Helena Kääriäinen, Markus Perola, Kati Kristiansson, Hanna Konttinen

**Affiliations:** ^1^ Social Psychology, Faculty of Social Sciences, University of Helsinki, Helsinki, Finland; ^2^ Motion Analysis Laboratory, Children’s Hospital, Helsinki University Hospital, Helsinki, Finland; ^3^ Research Program for Clinical and Molecular Metabolism, Faculty of Medicine, University of Helsinki, Helsinki, Finland; ^4^ Department of Public Health and Welfare, Finnish Institute for Health and Welfare, Helsinki, Finland

**Keywords:** polygenic risk, type 2 diabetes, coronary heart disease, Emotions, randomized controlled Trial

## Abstract

Receiving polygenic risk estimates of future disease through health care or direct-to-consumer companies is expected to become more common in the coming decades. However, only a limited number of studies have examined if such estimates might evoke an adverse psychosocial reaction in receivers. The present study utilized data from a sub-section of a personalized medicine project (the P5 study) that combines genomic and traditional health data to evaluate participants’ risk for certain common diseases. We investigated how communication of future disease risk estimates related to type 2 diabetes and coronary heart disease influenced respondents’ risk perception, self-efficacy, disease-related worry, and other emotions. A randomized controlled trial was conducted, where the experimental group (n = 714) received risk estimates based on traditional and polygenic risk factors and the control group (n = 649) based solely on traditional risk factors. On average, higher disease risk was associated with higher perceived risk (*p*s, <0.001, η_p_^2^ = 0.087–0.071), worry (*p*s <0.001, η_p_^2^ = 0.061–0.028), lower self-efficacy (*p* <0 .001, η_p_^2^ = 0.012), less positive emotions (*p*s <0.04, η_p_^2^ = 0.042–0.005), and more negative emotions (*p*s <0.048, η_p_^2^ = 0.062–0.006). However, we found no evidence that adding the polygenic risk to complement the more traditional risk factors would induce any substantive psychosocial harm to the recipients (*p*s >0.06).

## 1 Introduction

Type 2 diabetes (T2D) and coronary heart disease (CHD) affect millions of people globally and are significant contributors to disability and mortality worldwide (Sanchis-Gomar et al., 2016; Zheng, Ley, and Hu, 2018). The etiology of T2D and CHD indicates that, besides lifestyle-related risk factors, the risk of diabetes or heart disease is influenced by genetics. (Roberts and Stewart, 2012; Fuchsberger et al., 2016). Currently, genomic risk scores estimating the risk of future diseases such as diabetes or coronary heart disease are not routinely used in public health care. However, genotyping has become considerably cheaper and faster than in previous years. It is expected that genomic risk estimates will become increasingly more common in clinical and commercial contexts alike (Krier, Kalia and Green, 2016; Abul-Husn and Kenny, 2019).

However, it is not clear how receiving such risk information is beneficial in disease prevention or health behavior change. Most studies concerning genomic risk information’s effect on health behavior have produced nonsignificant or mixed results (e.g., Hollands et al., 2016; Peterson et al., 2018). Influence on psychosocial factors, such as emotional reaction the results, has been studied less extensively, and a considerable heterogeneity exists in the quality and methodology of the relevant studies (for a summary, see Wade, 2019; and for meta-analysis, see Robinson et al., 2019). Moreover, since a large part of the studies has focused on cancer-related testing, not much is known concerning future disease risk estimates influence in the context of other common diseases such as diabetes and cardiovascular disease.

Consequently, it would be relevant to expand the scope from cancer risk-related information to include other diseases. The field would also benefit from more large-scale experimental studies related to the psychosocial outcomes of genomic risk estimates. Thus, the current study aims to improve knowledge on how communication of genomic risk information related to type 2 diabetes and coronary heart disease influences respondents’ risk perception, self-efficacy, disease-related worry, and other emotions in the context of a randomized controlled trial. T2D and CHD were selected as suitable conditions for the current study since both are common diseases in Finland, and risk mitigation is possible through lifestyle changes (Abouzeid et al., 2015; Salomaa et al., 2016). Furthermore, genome-wide polygenic scores have been published for both conditions (Khera et al., 2018).

Self-efficacy (i.e., persons subjective estimate of the amount of control they expect to have in any given situation; Bandura, 1997), perception of disease risk, and emotional responses to risk information are concepts widely utilized in common health behavioral models and are known antecedents of health behavior (for example, see Hagger et al., 2020). Besides theoretical interest, there is also an apparent practical value in studying psychosocial outcomes. Suppose genomic risk estimates are to be more widely distributed in the context of common diseases. In that case, it is crucial to investigate that no active harm is caused to recipients as a byproduct, especially since the benefits of receiving such information remain uncertain.

So far, relatively few randomized controlled trials (RCT) have rigorously assessed the psychosocial impact of receiving actual genomic risk estimates related to T2D or CHD. A small-scale RCT by Grant et al. (2013) offered a diabetes prevention program to all participants and then compared effects on an experimental group supplemented with a genetic risk score for T2D and a control group, which received no risk score. No difference was found between the groups for risk perception, motivation, or confidence in diabetes-related lifestyle changes. In 2015, Voils et al. found no effect for perceived risk when a comparison was made between an experimental group receiving lifestyle and phenotypic and genomic risk estimate for T2D and a control group receiving lifestyle and phenotypic risk for T2D and risk score for unrelated eye disease. Likewise, a study by McVay et al. (2015), using the same sample, found no long-term effects regarding control perceptions or self-efficacy relating to diabetes. In 2016, Godino et al. examined the effects of receiving either standard lifestyle advice (control group) or a combination of lifestyle advice and a genomic or a phenotypic risk estimate for type 2 diabetes. The study found no effect regarding anxiety or worry respondents felt after the test results. The perception of risk for type 2 diabetes was lower in both intervention groups. However, no difference was found between genomic and phenotypic risk scores (see also Silarova et al., 2018 concerning the same sample). In relation to CHD, Robinson et al. (2016) investigated, among other things, how the reception of genomic risk score in addition to conventional risk estimate influenced participants’ perceived personal control when compared to a group receiving only conventional risk estimate. Out of the three subscales tested, the experimental group had slightly higher perceived cognitive control than participants receiving only conventional risk estimates.

To sum up, earlier studies have not reported adverse effects on receiving genomic risk information concerning T2D or CHD. However, only a limited number of studies have been conducted, especially related to CHD, and mainly cognitive outcomes have been examined. The present study aims to confirm earlier findings concerning risk perception and self-efficacy and expand from previous studies by investigating the emotional reaction to the test results. Moreover, since the size of the disease risk varies individually, it is also relevant to inspect if different risk levels cause different reactions depending on what type of risk information the participants have received. For example, it is conceivable that if one’s risk for T2D or CHD is low, it might matter less whether the risk was calculated based on genomic or more traditional factors. However, the same might not apply if one is at high risk for the disease. Thus, the specific research questions are:

1. Does receiving T2D- and CHD-related risk estimates based on a combination of genome-wide polygenic and traditional risk factors influence risk perception, self-efficacy, disease-related worry, or other emotions differently compared to receiving estimates based solely on traditional risk factors?
2. Is the magnitude of disease risk differently associated with the psychosocial factors depending on whether the respondent received estimates based on a combination of genome-wide polygenic and traditional risk factors or solely on traditional risk factors (i.e., is there an interaction between the disease risk level and type of risk information)?

## 2 Materials and Methods

### 2.1 Participants and Procedure

The present study utilized data from a sub-section of the P5 study^1^. P5 is a personalized medicine project led by the Finnish Institute for Health and Welfare. The project combines genomic and traditional health data to evaluate participants’ risk for certain common diseases and then utilizes an internet portal to return the future disease risk estimates (see Marjonen et al., 2021). The current study extracted data from two time points in 2019–2020. Namely, just before the respondents had access to their risk estimates of future disease concerning type 2 diabetes (T2D) and coronary heart disease (CHD) and after the participants had seen their estimates and filled the post-results survey. Access to the risk estimates was granted on 19.11.2019 for the experimental group and 16.12.2019 for the control group (all participants also received a reminder to fill in the surveys on 28.1.2020). Before participants could see their results, they had to fill in the pre-results survey. Most participants (over 90%) returned the post-results survey either on the same day as they filled the pre-results survey (34%) or within 76 days after filling the pre-results survey (56%). The possible confounding effect of response time was checked with a sensitivity analysis where the number of days each respondent had between returning the first and second survey was added as a covariate in the models. The sensitivity analysis resulted in only minute differences, see Supplementary File S1.

The future disease risk estimates were calculated based on respondents’ risk of developing T2D/CHD during the next 10-years. T2D risk was categorized to 4 levels (below 7.5% risk as low, 7.5–10% as elevated, 10–20% as high, and more than 20% as very high) and CHD risk in 3 levels (below 7.5% as low, 7.5–10% as elevated, and more than 10% as high). Participants also received information on selected single clinical variants (SCVs) related to CHD and venous thromboembolism (see Marjonen et al., 2021 for details). However, since the SCVs were not the main topic of the current study, respondents with one or more SCV were dropped from the final sample (n = 91). An exception was made regarding the pharmacogenetic variant of the *SCL O 1B1* gene, which was found abundantly in the current sample (n = 1189), but which did not influence the risk for CHD or venous thromboembolism. Participants with the *SCL O 1B1* variant were normally randomized to the experimental and the control group (N = 591 and 598, respectively).

The experimental group received disease risk estimates based on traditional risk factors and genome-wide polygenic scores (GPS), and the control group received estimates based solely on the traditional risk factors. Traditional risk factors included: BMI, total cholesterol, high-density lipoprotein, systolic blood pressure, blood pressure-lowering medication, lipid-lowering medication, self-reported family history, and smoking status. We used GPSes, previously published in the UK Biobank population, containing close to 7 million genomic variants for T2D and 6.6 million variants for CHD (Khera et al., 2018). To calculate the GPSes, all variants were collected from the imputed data and weighted by their corresponding genotype effect sizes. Imputed data contained 94% of the original variants in the T2D and CHD polygenic scores. After summing the variants together, the GPS was standardized using the mean and standard deviation (SD) of the GPS in an independent population sample. If a variant was missing from the imputed data (missingness 0.05%), the population average frequency of the genotype was used in the calculation instead.

The risk scores were presented as a number, accompanied with a short-written description of the risk score, and as a graph where participant’s risk was contrasted to the above-mentioned risk levels for the relevant disease (see Marjonen et al., 2021 for examples). In addition to the 10-years combined disease risk, the experimental group was provided with a graph showing their genome-wide polygenic risk scores separately. Also, a health report was given to all participants, which gave a more thorough explanation of the results and provided personal instructions on how the participants could influence their disease risk with lifestyle changes (see Marjonen et al., 2021). Finally, an interactive calculator was introduced, where respondents could test how changing their physical parameters (e.g., age) and lifestyle factors (e.g., smoking) would change their risk score (see Marjonen et al., 2021).

**FIGURE 1:**
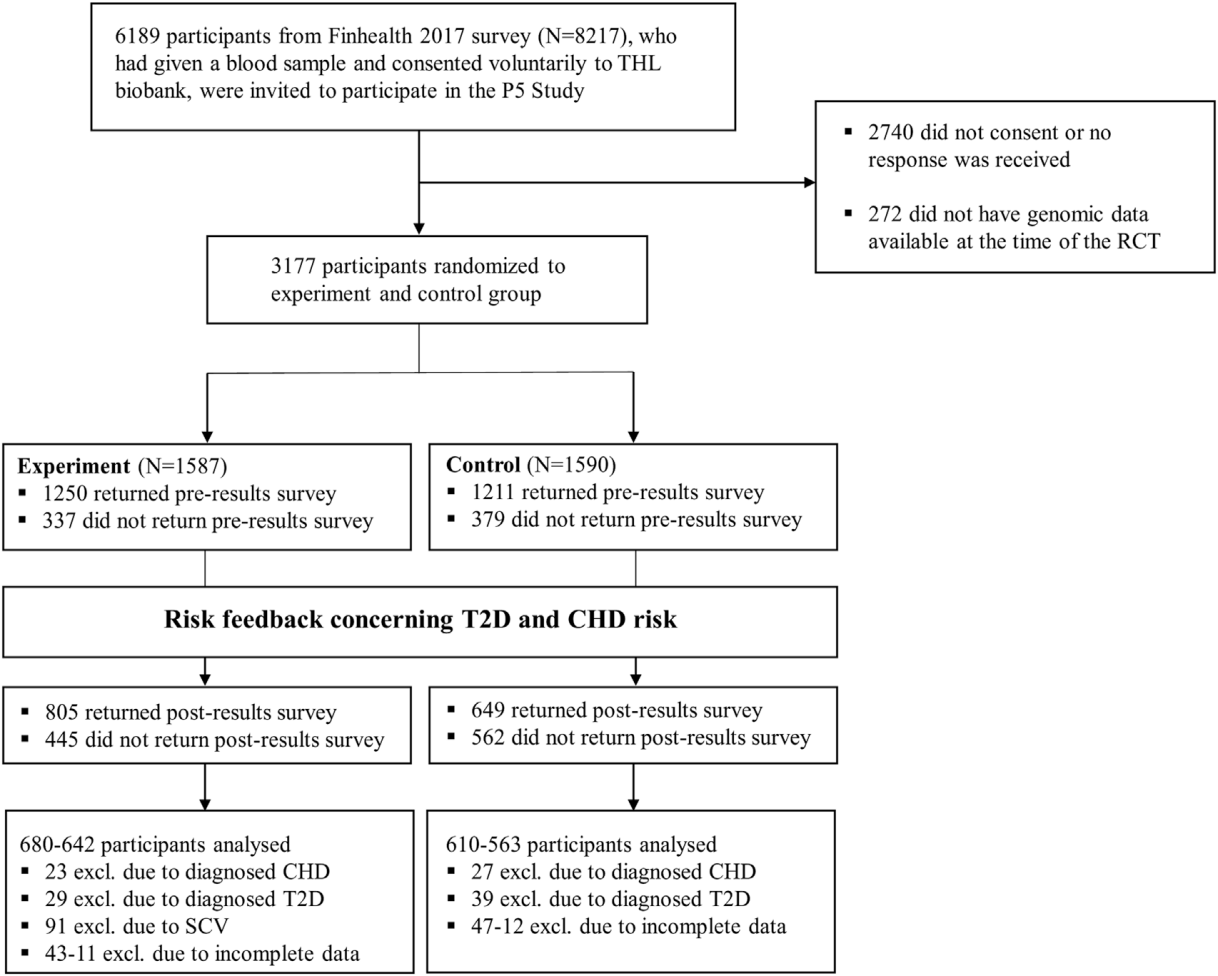
Flowchart for the current study.

In total, 3449 respondents (drawn from a population-based survey of 8217; see Marjonen et al., 2021) consented to participate in the P5 study, and 3177 had risk results given to them (272 participants did not have genomic data available at the time of the RCT). 2290 respondents returned the pre-results survey, and 1368 out of 2290 the post-results survey (see Table 1 and Figure 1). To more formally access attrition, respondents who dropped (i.e., those randomized to experimental/control group but who did not return the post-survey; n = 1809) and who returned the post-survey (n = 1368) were compared with each other. The chi-square test of independence indicated that respondents who stayed and who dropped differed in terms of all the socio-demographic variables (*p*s < 0.03). However, the association’s size was relatively small in all cases (Cramer’s V = 0.04–0.21). Noteworthy, respondents who dropped were somewhat older (V = 0.21), less educated (V = 0.20), more likely to be pensioners (V = 0.16), had lower income (V = 0.17), and had a higher risk for CHD (V = 0.19). (See Supplementary File S1 for full results and Marjonen et al., 2021 for comparison of the population-based survey and the P5 study.).

**TABLE 1:** Sample descriptives at three time points, and between the experimental and the control group after the post-results survey.

		After Randomization		Pre-results		Post-results		Experimental/Control group	
N Total (Per Condition)		3177 (1587/1590)		2290 (1079/1211)		1368 (714/649)		1368 (714/649)	
Female (%)		56		57		58		56/61	
Age group (%)									
<30		7		8		8		9/6	
30–39		13		16		14		14/14	
40–49		15		18		18		19/17	
50–59		22		24		25		26/24	
60–69		26		24		26		24/28	
70–79		14		9		8		7/9	
>80		3		1		1		1/1	
Educational level (%)									
comprehensive		15		11		9		8/10	
intermediate		33		31		29		30/27	
university		52		59		62		62/62	
Occupational status (%)									
employed		52		60		61		64/57	
unemployed		5		5		5		4/5	
student		3		4		4		3/4	
pensioner		36		28		28		25/31	
Other		3		3		3		3/4	
Annual income (%)									
25000 € or less		19		14		13		14/13	
25001–45000 €		27		25		23		23/24	
45001–60000 €		19		20		21		22/21	
60001–80000 €		17		19		19		18/21	
over 80000 €		18		22		23		24/21	
Marital status (%)									
has a partner		74		76		75		75/76	
single		11		12		12		12/12	
divorcee or widow		15		13		13		14/12	
Has no children (%)		21		23		24		25/23	
Risk level categories (%)		T2D	CHD	T2D	CHD	T2D	CHD	T2D	CHD
low		70	71	73	78	73	80	75/72	81/80
elevated		8	8	7	6	7	6	7/7	5/6
high		14	22	12	16	12	14	11/12	15/14
very High		8		7		8		7/8	

Note. the mean and standard deviation for GPS, based risk category (1–6) for the experimental group is: After randomization, T2D M = 2.97 (SD, 0.7), CHD M = 2.99 (SD, 0.72); Pre-results, T2D M = 2.98 (SD, 0.71), CHD M = 2.98 (SD, 0.72); Post-results, T2D M = 2.97 (0.72), CHD M = 2.96 (SD, 0.73). Where GPS, Category 1 refers to GPS, 1.96 SD, or more below the mean; Category 2 to 1.96–0.84 SD, below the mean; Category 3 to 0.84–0 SD, below the mean; Category 4 to 0–0.84 SD, over the mean; Category 5 to 0.84–1.96 SD, over the mean; and Category 6 to 1.96 SD, or more over the mean.

Before the analyses were carried out, respondents with T2D already diagnosed (n = 68) or CHD already diagnosed (n = 50) were excluded from the relevant models (see Analysis and design). After listwise deletion was used, the final models included 1202–1290 respondents.

Moreover, since the number of available participants determined the sample size, no a priori power analysis could be done. Instead, a sensitivity power analysis for the two statistical models intended to be used, namely analysis of covariance (ANCOVA) and analysis of variance (ANOVA), was conducted with the (smallest) available sample size. G*Power software (Faul et al., 2007) indicated that when ANCOVA (n = 1231) with 1 covariate and 2 × 4 factorial design was used, the smallest effect for interaction that could be detected with 0.05 alpha and 80% power was Cohen’s f = 0.094. Similarly, for 2 × 4 factorial ANOVA (n = 1202) the smallest effect for interaction that could be detected with 0.05 alpha and 80% power was Cohen’s f = 0.095.

### 2.2 Measures

#### 2.2.1 Perceived Risk

Perceived risk for T2D and CHD was measured with one item (per disease) before and after the risk scores were released. The wording was identical for both diseases: “How do you perceive your risk for T2D/CHD?” where 1 was “very small,” and 5 was “very large.”

#### 2.2.2 Self-Efficacy

Similarly, self-efficacy was measured with one item before and after the risk scores were released: “To what extent do you feel that you can influence whether you develop T2D/CHD”, where 1 was “Not at all,” and 7 was “Very much.”

#### 2.2.3 Risk-Related Worry

Different questions were used for risk-related worry in the pre- and post-results questionnaires. Before the risk scores were released, participants’ *general worry* of T2D/CHD risk was measured with one item: “How worried you are concerning your risk for T2D/CHD?”. Then, after the risk scores were released, separate items were used for worry related to *genetic factors* and worry related to *traditional risk factors*: “How worried you are concerning your risk for T2D/CHD based on “traditional” (e.g., cholesterol)/genetic risk factors.” That is, the pre-results questionnaire included two worry related questions (one for T2D and one for CHD), and the post-results questionnaire included four worry related questions (worry related to genetic risk/traditional risk factors for TD2/CHD). Each item was rated from 1 “Not at all” to 7 “Very much.”

#### 2.2.4 Emotional Reactions (MICRA)

Participants’ immediate emotional reaction to the test results was measured with eight items selected from the Multidimensional Impact of Cancer Risk Assessment (MICRA; Cella et al., 2002) questionnaire, a well-established scale used to monitor patient concerns related to genetic testing for cancer. The selected items (items specific to cancer testing were excluded) reflected a range of emotional reactions respondents could experience due to their test results. The following items were included: “Feeling upset about my test result,” “Feeling sad about my test result,” “Feeling anxious or nervous about my test result,” “Feeling guilty about my test result,” “Feeling relieved about my test result,” “Feeling happy about my test result,” “Feeling a loss of control,” and “Having problems enjoying life because of my test result.” All items were rated from 1 (“Very little or not at all”) to 5 (“Very much”).

MICRA subscales were not utilized. The reason for this was twofold; for one, not all MICRA items were included in the study (e.g., cancer-specific items were excluded), making it difficult to estimate the proper aggregate scores. Secondly, we wanted to explore the diversity of people’s emotional reactions to polygenic risk scores, not just changes in more general emotional dispositions that MICRA subscales depict (e.g., positive/negative reaction).

See Marjonen et al. (2021) for all the scales used in the study.

### 2.3 Analysis and Design

Since the MICRA scale was used only after the respondents had seen their test results (as these questions specifically measured participants *reaction to the test results*) and all other items had an appropriate baseline score measured before the risk scores were released, different statistical models were applied to the MICRA items and the other dependent variables. Consistent with the sensitivity power analysis, a 0.05 alpha level was adopted for all statistical tests. All analyses were carried out with RStudio v1.4.1717.

#### 2.3.1 Analysis of Perceived Risk, Self-Efficacy, and Risk-Related Worry

For a simple pretest-posttest design, ANCOVA is the typically preferred method (Huitema, 2011) and has shown to perform well in comparison to many alternatives (e.g., Egbewale et al., 2014; Zhang et al., 2014; Chausse et al., 2016; O'Connell et al., 2017). Thus, an ANCOVA model was selected for all the dependent variables with an appropriate baseline score. More specifically, a 2-way ANCOVA with the type of risk information received (only traditional/combination of traditional and genetic) and risk level (low, elevated, high, and very high for T2D, or low, elevated, and high for CHD) as factors, and score of the dependent variable in the pre-results survey as a covariate was used for each model. Dependent variables included perceived risk for T2D/CHD, worry concerning one’s risk for T2D/CHD based on traditional/genetic factors, and self-efficacy concerning one’s ability to prevent the development of T2D/CHD. In case of significant main effect, a pairwise comparison with estimated marginal means was implemented to test which groups differed from each other formally. If a significant interaction emerged, it was first plotted and eyeballed, and then pairwise comparisons were carried out for each factor within the levels of the other factor. Type 3 sums of squares were used in all ANCOVA models, as is recommended for designs where interaction between the factors is inspected (Huitema, 2011). Moreover, since multiple tests were performed, a Holm correction was applied in all ANCOVA models and in subsequent comparisons to assure appropriate control for the Type I error rate (Abdi, 2007). Partial eta squared (*η*p2) was used as an effect size estimator for the F-test and Cohen’s d for pairwise comparisons of estimated marginal means (both estimated from the relevant test statistic and degrees of freedom).

#### 2.3.2 Analysis of Emotional Reactions (MICRA)

As mentioned before, no covariate was available for the MICRA scale. Thus, an ANOVA model with each MICRA item as a dependent variable was used instead of the ANCOVA model. When significant effects emerged, a similar approach was utilized as described earlier with the ANCOVA models (i.e., Holm correction, pairwise comparison of marginal means, and so on). Moreover, since the MICRA items were not written in relation to either of the disease risks but asked solely how the respondents felt about their “test results,” it was not a priori clear if separate ANOVAs should be calculated for each disease risk or if both risks should be included in the same model. That being the case, it was first tested if any significant three-way interactions concerning the type of risk information received and T2D and CHD risk level would emerge (see Supplementary File S1 for three-way ANOVA results). When none was found, it was decided that for simplicity, each MICRA item would be analyzed with two 2-way ANOVAs. Once with T2D risk level and once with CHD risk level as a factor.

## 3 Results

### 3.1 Perceived Risk for T2D and CHD

Two separate 2-way ANCOVAs were calculated for perceived risk related to T2D and CHD. In both models, perceived risk, measured in the post-results survey, was used as a dependent variable, perceived risk in the pre-results survey a covariate, and participant’s risk level (low, elevated, high, and very high for T2D; and low, elevated, and high for CHD) and the type of risk information received (combination of genetic and traditional or traditional only) as factors. As can be seen from Table 2, risk level had a significant main effect in both disease conditions (both *p*s <0.001), but no significant effect was found for the type of risk information given or for the interaction between risk level and type of information (*p*s >0.12).

**TABLE 2:** Analysis of covariance main effects and interactions: Perceived risk, self-efficacy, and worry concerning T2D and CHD risk.

T2D								CHD					
Dependent	Effect	Marginal means (SE)		F	df	*p*	η_p_^2^	Marginal means (SE)		F	df	*p*	η_p_^2^
Perceived risk	Risk type	G + T	2.56 (0.05)	2.26	1, 1242	0.13	0.002	G + T	2.51 (0.05)	0.24	1, 1283	0.62	<0.001
		T	2.45 (0.06)					T	2.48 (0.05)				
	Risk level	Low	2.11 (0.02)^A^	31.69	3, 1242	<0.001	0.071	Low	2.18 (0.02)^A^	60.82	2, 1283	<0.001	0.087
		Elevated	2.54 (0.07)^B^					Elevated	2.55 (0.09)^B^				
		High	2.62 (0.06)^B^					High	2.75 (0.05)^C^				
		Very High	2.76 (0.12)^B^										
	Risk type × Risk level			1.95	3, 1242	0.12	0.005			0.39	2, 1283	0.69	0.001
Self-efficacy	Risk type	G + T	5.51 (0.08)	<0.01	1,1248	0.96	<0.001	G + T	5.22 (0.08)	0.11	1, 1262	0.74	<0.001
		T	5.51 (0.08)					T	5.18 (0.08)				
	Risk level	Low	5.64 (0.03)	1.75	3, 1248	0.16	0.004	Low	5.42 (0.03)^A^	7.95	2, 1262	<0.001	0.012
		Elevated	5.47 (0.12)					Elevated	5.11 (0.14)^B^				
		High	5.47 (0.10)					High	5.07 (0.10)^B^				
		Very High	5.46 (0.17)										
	Risk type × Risk level			1.95	3, 1248	0.12	0.005			4.64	2, 1262	0.01	0.007
Worry related to traditional risk	Risk type	G + T	3.48 (0.09)	0.01	1, 1248	0.92	<0.001	G + T	3.67 (0.09)	3.52	1, 1278	0.06	0.003
		T	3.50 (0.09)					T	3.43 (0.09)				
	Risk level	Low	2.83 (0.04)^A^	26.90	3, 1248	<0.001	0.061	Low	3.13 (0.04)^A^	18.56	2, 1278	<0.001	0.028
		Elevated	3.48 (0.15)^B^					Elevated	3.85 (0.16)^B^				
		High	3.67 (0.11)^B^					High	3.66 (0.10)^B^				
		Very High	3.98 (0.19)^B^										
	Risk type × Risk level			0.19	3, 1248	0.90	<0.0.001			3.42	2, 1278	0.03	0.005
Worry related to genetic risk	Risk type	G + T	3.23 (0.10)	0.11	1, 1226	0.75	<0.001	G + T	3.44 (0.11)	0.04	1, 1256	0.85	<0.001
		T	3.27 (0.11)					T	3.41 (0.09)				
	Risk level	Low	2.66 (0.04)^A^	19.21	3, 1226	<0.001	0.045	Low	2.94 (0.05)^A^	22.99	2, 1256	<0.001	0.035
		Elevated	3.18 (0.17)^B^					Elevated	3.68 (0.18)^B^				
		High	3.45 (0.13)^B^					High	3.65 (0.11)^B^				
		Very High	3.71 (0.19)^B^										
	Risk type × Risk level			0.84	3, 1226	0.47	0.002			1.74	2, 1256	0.18	0.003

Note. Heteroskedasticity consistent covariate matrix HC3 is used in all models. *p*-values are Holm adjusted in each model. Means that do not share a letter are significantly different (with 0.05 alpha level). Partial eta squared is estimated based on degrees of freedom and F-values.

Estimated marginal means and pairwise comparisons were calculated to investigate the significant main effects further. When perceived risk related to T2D was inspected, participants with low risk also perceived their risk as lower compared to respondents with elevated (MD = 0.43 ± 0.08, d = 0.32), high (MD = 0.52 ± 0.07, d = 0.44), or very high (MD = 0.65 ± 0.13, d = 0.30) risk (*p*s <0.001). However, no differences were found when participants with elevated, high, or very high risk were compared to each other (*p*s > 0.34). A somewhat similar pattern emerged regarding perceived risk related to CHD. As before, respondents with low risk perceived their risk lower compared to participants with elevated or high risk (both *p*s <0.001, MD = 0.36–0.57 ± 0.09–0.05, d = 0.22–0.59). In addition, participants with elevated risk also perceived their risk lower compared to participants with high risk (*p* = 0.04, MD = 0.21 ± 0.10, d = 0.11).

### 3.2 Self-Efficacy Concerning One’s Ability to Prevent T2D and CHD

Similar 2-way ANCOVAs as described above were calculated concerning self-efficacy. As before, the type of risk information and level worked as factors, and self-efficacy score before the risk information was released was used as a covariate. In relation to self-efficacy and T2D, no statistically significant main effects or interaction emerged for the type of information received or for risk level (*p*s > 0.12). When CHD and self-efficacy were inspected, a nonsignificant main effect for type of risk (*p* = 0.74) and a significant main effect for risk level (*p* = <0.001) emerged. Moreover, an interaction between the type of risk information and risk level was found (*p* = 0.01).

**FIGURE 2:**
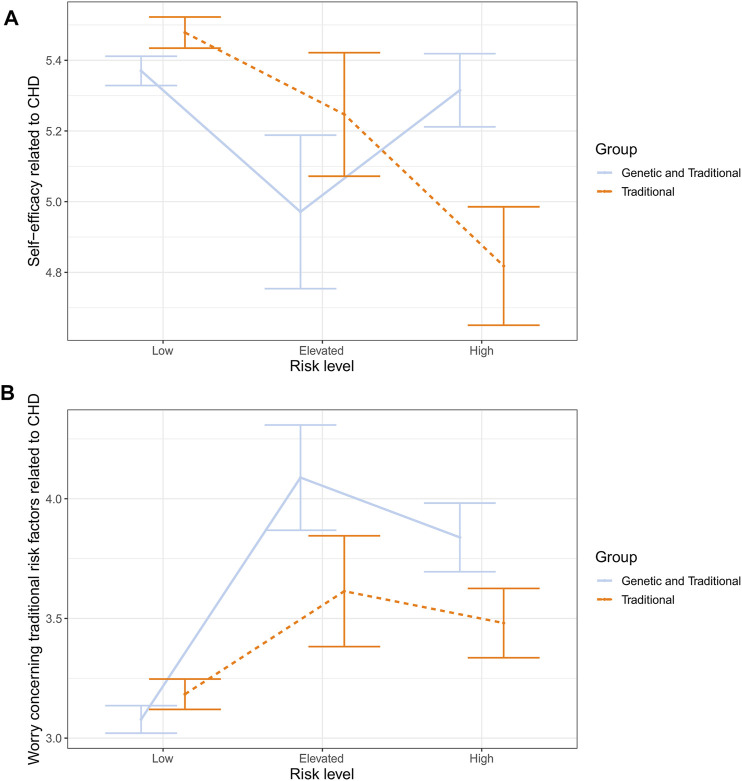
Estimated means and standard errors for interaction between risk type and risk level concerning CHD related self-efficacy **(A)** and worry **(B)**.

Since the interaction effect was statistically significant, the main effect of risk level was not further investigated (see Table 2 for main effect results). Instead, the interaction between the risk level and type of information was plotted and eyeballed (see Figure 2A). Then pairwise comparisons were carried out for each factor within the levels of the other factor (i.e., low vs. elevated vs. high CHD risk comparisons made separately for both types of risk, and type of risk comparisons made separately within each CHD risk level). Within the group receiving only traditional risk information, it seemed that higher risk was associated consistently with lower self-efficacy. Pairwise comparisons indicated differences between respondents with low and high risk (*p* < 0.001, MD = 0.66 ± 0.17, d = 0.21), but not for other risk levels (*p*s > 0.15). As for the group receiving a combination of genetic and traditional risk, the plot indicated a V-shape trend: low- and high-risk participants seemed to have almost equal self-efficacy while respondents with elevated risk had relatively reduced self-efficacy. However, none of the differences reached statistical significance when pairwise comparisons were carried out (*p*s > 0.21).

When the effect of type of risk was, in turn, inspected in the classes of risk level, pairwise comparisons indicated that the group receiving a combination of traditional and genetic information had, compared to the control group, higher self-efficacy within the high-risk level (*p* = 0.01, MD = 0.50 ± 0.20, d = 0.14), while no difference was found within low or elevated risk levels (ps > 0.07).

### 3.3 Worry Related to T2D and CHD Risk Based on Traditional Risk Factors

Two separate two-way ANCOVAs were conducted. General worry concerning T2D/CHD measured in the pre-results survey was used as a covariate, and worry concerning traditional risk for T2D/CHD measured in the post-results survey was used as a dependent variable. Again, the type of risk and risk level worked as factors in both models. In relation to worry concerning traditional risk for T2D, no significant main effect was found for the type of risk information received (*p* = 0.92) or for the interaction between the risk type and risk level (*p* = 0.90). However, the risk level did have a significant main effect (*p* < 0.001). Likewise, in relation to CHD, no significant main effect was found for the type of risk information (*p* = 0.06). Significant effects did, however, emerge for the risk level (*p* <0 .001) and for the risk type and level interaction (*p* = 0.03).

Pairwise comparisons indicated that respondents with low risk for T2D were significantly less worried than participants who had elevated (MD = 0.64 ± 0.15, d = 0.24), high (MD = 0.84 ± 0.12, d = 0.39), or very high (MD = 1.44 ± 0.19, d = 0.34) risk for T2D (*p*s < 0.001). However, no differences were found when elevated-, high-, and very high-risk groups were compared to each other (*p*s > 0.10).

In relation to CHD, due to the significant interaction, the main effect of the risk level was not further investigated (see Table 2 for main effect results). As before, the interaction was first plotted and eyeballed, and then pairwise comparisons were made. Both the experiment and the control group showed a roughly similar pattern of results (see Figure 2B). CHD-related worry was the lowest at a low-risk level, then spiked up at an elevated risk level, and slightly dropped at a high-risk level. Although roughly similar, the pattern seemed more pronounced in the group receiving traditional and genetic risk information. However, when pairwise comparisons were conducted comparing the two groups at different risk levels, no differences emerged at any stage (all *p*s > 0.08). The only statistically significant differences emerged within the group receiving traditional and genetic risk information. Here, low-risk respondents were less worried than elevated- (MD = 1.01 ± 0.23, d = 0.25) or high-risk (MD = 0.76 ± 0.16, d = 0.27) participants (both *p*s <0.001).

### 3.4 Worry Related to T2D and CHD Risk Based on Genetic Risk Factors

Similar two-way ANCOVAs as described above were calculated for worry related to genetic risk factors. As before, type of risk and risk level worked as factors, and general worry related to T2D/CHD measured in the pre-results survey was used as a covariate. No significant main effects were found in either disease condition for the type of risk information received (*p*s > 0.75) or for the interaction between the type of risk and risk level (*p*s > 0.18). However, risk level did have a significant main effect in both disease conditions (both *p*s < 0.001).

Pairwise comparisons revealed that participants with low risk for T2D were less worried about their T2D risk related to genetic risk factors than respondents with elevated (MD = 0.53 ± 0.17, d = 0.17), high (MD = 0.79 ± 0.14, d = 0.33), or very high (MD = 1.05 ± 0.20, d = 0.30) risk (*p*s < 0.01). However, no significant differences were observed between elevated-, high-, and very high-risk groups (*p*s > 0.12). When the risk for CHD was inspected, participants with low risk were less worried concerning their risk related to genetic factors than respondents with elevated (MD = 0.74 ± 0.19, d = 0.22) or high (MD = 0.71 ± 0.11, d = 0.34) risk (*p*s < 0.001). Again, no difference emerged between elevated- and high-risk groups (*p* = 0.90).

### 3.5 Emotional Reactions to Test Results (MICRA)

Separate 2-way ANOVA models were conducted for the MICRA items. All models included the type of risk and risk level as factors. The analyses were carried out once with T2D risk level as a factor and once with CHD risk level as a factor (see Table 3).

**TABLE 3:** Analysis of variance main effects and interactions: Emotional reaction to the test results concerning T2D and CHD risk.

T2D								CHD					
Dependent	Effect	Marginal means (SE)		F	df	*p*	η_p_^2^	Marginal means (SE)		F	df	*p*	η_p_^2^
Upset	Risk type	G + T	1.60 (0.06)	0.95	1, 1203	0.33	0.001	G + T	1.56 (0.06)	0.83	1, 1227	0.36	0.001
		T	1.52 (0.06)					T	1.48 (0.06)				
	Risk level	Low	1.17 (0.02)^A^	26.36	3, 1203	<0.001	0.062	Low	1.24 (0.02)^A^	26.36	2, 1227	<0.001	0.041
		Elevated	1.57 (0.10)^B^					Elevated	1.59 (0.11)^B^				
		High	1.60 (0.07)^B^					High	1.71 (0.07)^B^				
		Very High	1.88 (0.12)^B^										
	Risk type × Risk level			0.29	3, 1203	0.83	0.001			1.95	2, 1227	0.14	0.003
Sad	Risk type	G + T	1.55 (0.06)	0.33	1, 1201	0.57	<0.001	G + T	1.52 (0.06)	0.14	1, 1225	0.71	<0.001
		T	1.60 (0.06)					T	1.49 (0.06)				
	Risk level	Low	1.17 (0.02)^A^	13.58	3, 1201	<0.001	0.033	Low	1.24 (0.02)^A^	20.48	2, 1225	<0.001	0.032
		Elevated	1.56 (0.10)^B^					Elevated	1.56 (0.11)^B^				
		High	1.68 (0.07)^B^					High	1.73 (0.07)^B^				
		Very High	1.91 (0.11)^B^										
	Risk type × Risk level			0.78	3, 1201	0.50	0.002			1.43	2, 1225	0.24	0.002
Nervous	Risk type	G + T	1.47 (0.06)	0.74	1, 1199	0.39	0.001	G + T	1.37 (0.05)	0.01	1, 1223	0.91	<0.001
		T	1.41 (0.05)					T	1.36 (0.05)				
	Risk level	Low	1.17 (0.02)^A^	10.13	3, 1199	<0.001	0.025	Low	1.22 (0.02)^A^	9.04	2, 1223	<0.001	0.015
		Elevated	1.45 (0.09)^B^					Elevated	1.34 (0.08)^AB^				
		High	1.45 (0.07)^B^					High	1.54 (0.06)^B^				
		Very High	1.71 (0.10)^B^										
	Risk type × Risk level			0.88	3, 1199	0.45	0.002			0.71	2, 1223	0.49	0.001
Guilt	Risk type	G + T	1.60 (0.06)	<0.01	1, 1201	0.99	<0.001	G + T	1.43 (0.06)	0.80	1, 1224	0.37	0.001
		T	1.60 (0.06)					T	1.50 (0.06)				
	Risk level	Low	1.19 (0.02)^A^	14.53	3, 1201	<0.001	0.035	Low	1.28 (0.02)^A^	8.90	2, 1224	<0.001	0.014
		Elevated	1.66 (0.10)^BC^					Elevated	1.44 (0.10)^AB^				
		High	1.57 (0.08)^B^					High	1.69 (0.07)^B^				
		Very High	1.97 (0.12)^C^										
	Risk type × Risk level			1.33	3, 1201	0.26	0.003			0.09	2, 1224	0.91	<0.001

T2D								CHD					
Dependent	Effect	Marginal means (SE)		F	df	*p*	η_p_^2^	Marginal means (SE)		F	df	*p*	η_p_^2^
Relieved	Risk type	G + T	3.00 (0.07)	0.36	1, 1207	0.55	<0.001	G + T	3.08 (0.08)	0.02	1, 1231	0.89	<0.001
		T	3.06 (0.08)					T	3.07 (0.09)				
	Risk level	Low	3.32 (0.04)^A^	6.59	3, 1207	<0.001	0.016	Low	3.26 (0.04)^A^	3.21	2, 1231	0.04	0.005
		Elevated	3.15 (0.12)^AB^					Elevated	3.00 (0.15)^AB^				
		High	2.87 (0.10)^B^					High	2.96 (0.09)^B^				
		Very High	2.79 (0.13)^B^										
	Risk type × Risk level			0.99	3, 1207	0.39	0.002			0.04	2, 1231	0.96	<0.001
Happy	Risk type	G + T	3.03 (0.08)	0.08	1, 1203	0.78	<0.001	G + T	3.15 (0.08)	0.42	1, 1227	0.52	<0.001
		T	3.00 (0.08)					T	3.07 (0.10)				
	Risk level	Low	3.61 (0.04)^A^	17.47	3, 1203	<0.001	0.042	Low	3.52 (0.04)^A^	14.79	2, 1227	<0.001	0.024
		Elevated	3.02 (0.13)^B^					Elevated	3.00 (0.16)^B^				
		High	2.81 (0.10)^B^					High	2.81 (0.09)^B^				
		Very High	2.64 (0.14)^B^								
	Risk type × Risk level			0.24	3, 1203	0.87	0.001	0.09	2, 1227	0.92	<0.001		
Loss of control	Risk type	G + T	1.15 (0.03)	0.50	1, 1200	0.48	<0.001	G + T	1.14 (0.03)	0.47	1, 1223	0.49	<0.001
		T	1.12 (0.03)					T	1.17 (0.04)				
	Risk level	Low	1.07 (0.01)^A^	2.65	3, 1200	0.048	0.007	Low	1.07 (0.01)^A^	5.57	2, 1223	0.004	0.009
		Elevated	1.08 (0.03)^AB^					Elevated	1.17 (0.07)^AB^				
	High	1.14 (0.04)^AB^	High					1.22 (0.04)^B^					
	Very High	1.26 (0.07)^B^											
	Risk type × Risk level					2.10	3, 1200	0.10	0.005	0.70	2, 1223	0.50	0.001
Difficulties enjoying life	Risk type	G + T	1.18 (0.04)	1.09	1, 1197	0.30	0.001	G + T	1.13 (0.03)	0.05	1, 1220	0.82	<0.001
		T	1.12 (0.03)					T	1.12 (0.03)				
	Risk level	Low	1.05 (0.01)^A^	3.14	3, 1197	0.03	0.008	Low	1.06 (0.01)^A^	3.86	2, 1220	0.02	0.006
		Elevated	1.08 (0.03)^A^					Elevated	1.09 (0.04)^A^				
		High	1.11 (0.04)^AB^					High	1.23 (0.05)^B^				
		Very High	1.37 (0.09)^B^										
	Risk type × Risk level			1.05	3, 1197	0.37	0.003			0.26	2, 1220	0.77	<0.001

Note. Heteroskedasticity consistent covariate matrix HC3 is used in all models. *p*-values are Holm adjusted in each model. Means that do not share a letter are significantly different (with 0.05 alpha level). Partial eta squared is estimated based on degrees of freedom and F-values.

Type of risk had a nonsignificant main effect in all models (*p*s > 0.30). Likewise, no statistically significant interaction between risk type and the risk level emerged concerning T2D risk (*p*s > 0.10) or CHD risk (*p*s > 0.14). However, a significant main effect did emerge for both CHD and T2D risk levels in all models (*p*s < 0.048).

As with the ANCOVA models, pairwise comparisons of estimated marginal means were carried out for the statistically significant effects. For the T2D risk level, the comparisons indicated a similar pattern of results concerning how upset, sad, nervous, or happy the respondents felt. Participants with low T2D risk were less upset (MD = 0.39–0.71 ± 0.10–0.12, d = 0.23–0.34), sad (MD = 0.40–0.52 ± 0.12–0.08, d = 0.22–0.39), nervous (MD = 0.28–0.54 ± 0.09–0.10, d = 0.18–0.30), and happier (MD = 0.60–0.80 ± 0.14–0.11, d = 0.25–0.43) than respondents with elevated, high, or very high risk (*p*s < 0.01). An almost similar pattern emerged concerning how much guilt the respondents felt due to their test results. Low-risk participants felt less guilt than respondents with elevated (MD = 0.47 ± 0.10, d = 27), high (MD = 0.38 ± 0.08, d = 0.28), or very high (MD = 0.78 ± 0.12, d = 37) risk (*p*s < 0.001). However, a statistically significant difference also emerged between respondents with high and very high risk (*p* = 0.02, MD = 0.40 ± 0.14, d = 0.16). Other differences related to upset, sadness, nervousness, guilt, happiness, and T2D risk level were statistically nonsignificant (*p*s > 0.07).

The amount of relief participants felt after seeing their results followed largely the same general pattern of the low-risk group producing the significant effects. However, no statistically significant effect was observed when low- and elevated-risk groups were compared to each other (*p* = 0.35, while other comparisons to low risk were again *p* < 0.001, MD = 0.54–0.46 ± 0.14–0.11, d = 0.22–0.24). When feelings of losing control were inspected, participants with low T2D risk felt more in control than participants with very high risk (*p* = 0.04, MD = 0.20 ± 0.07, d = 0.16). All other comparisons related to losing control were nonsignificant (*p*s > 0.07). Finally, when comparisons related to difficulties enjoying life were conducted, respondents with very high T2D risk had more difficulties compared to participants with low (MD = 0.32 ± 0.10, d = 0.20) or elevated (MD = 0.29 ± 0.10, d = 0.17) risk (*p*s < 0.02). All other comparisons were statistically nonsignificant (*p*s > 0.05).

Pairwise comparisons related to CHD risk and emotions produced reasonably similar results. Participants with low CHD risk were less upset (MD = 0.35–0.47 ± 0.11–0.07, d = 0.18–0.38), sad (MD = 0.32–0.49 ± 0.12–0.07, d = 0.16–0.40), and happier (MD = 0.52–0.72 ± 0.17–0.10, d = 0.18–0.41) than respondents with elevated or high risk (*p*s < 0.012), while all other differences were statistically nonsignificant (*p*s > 0.24). Likewise, respondents with low CHD risk felt less nervous (MD = 0.33 ± 0.07, d = 0.28), guilty (MD = 0.42 ± 0.07, d = 0.33), and loss of control (MD = 0.15 ± 0.04, d = 0.21) compared to respondents with high risk (*p*s < 0.01). However, no difference was found when low and elevated or high and elevated risk participants were compared to each other (*p*s > 0.07). Finally, when risk groups were compared in relation to how much difficulties they have enjoying life, respondents with low (MD = 0.17 ± 0.05, d = 0.20) or elevated (MD = 0.15 ± 0.06, d = 0.14) risk had less difficulties compared to high-risk participants (*p*s < 0.033), while no statistically significant difference emerged between participants with low and elevated risk (*p* = 0.54).

## 4 Discussion

An RCT was utilized to investigate the psychosocial effects of receiving a combination of traditional risk information and a genome-wide polygenic risk score concerning two common diseases. The experimental group was given a risk estimate related to type 2 diabetes (T2D) and coronary heart disease (CHD) based on a combination of genome-wide polygenic risk scores and traditional risk factors. The control group received risk estimates for the same diseases based solely on traditional risk factors. Afterward, the groups were compared in relation to perceived risk, self-efficacy, risk-related worry, and emotional reactions to the test results.

Type of risk (i.e., whether the respondents received future disease risk estimates based on a combination of genome-wide polygenic and traditional risk factors or traditional risk factors only) did not have a significant main effect in any comparisons made. Thus, our results are largely compatible with earlier studies concerning polygenic estimates of T2D and CHD risk, and polygenic risk scores more generally (e.g., Godino et al., 2016; Robinson et al., 2016; Robinson et al., 2019). Receiving a genome-wide polygenic risk in addition to more traditional risk factors for T2D or CHD does not, on average, seem to stir detrimental or any other kind of pronounced psychological reaction in the receivers when compared to receiving information based only on traditional risk factors.

However, two significant interaction effects were found when the type of risk information received was inspected alongside the risk level (i.e., how high respondents’ risk for T2D/CHD was). In relation to CHD risk and self-efficacy, the control group showed a linearly decreasing trend in self-efficacy as CHD risk increased. In the experimental group, low- and high-risk participants had very similar levels of self-efficacy, and participants with elevated risk had slightly lower self-efficacy. Multiple reasons could contribute to the observed pattern. For example, it has been suggested that genetic risk estimates might induce fatalistic beliefs concerning one’s chances of coming down with a certain disease (Claassen et al., 2010), which, in turn, might lead to insensitivity to actual risk.

However, empirical evidence that polygenic risk scores would inflict fatalistic beliefs in real-life situations has been scant (Collins et al., 2011). Moreover, at least at first glance, it seems peculiar that no such interaction was found for T2D risk and self-efficacy. Though, it is possible that *the knowledge of not having* certain CHD risk increasing SCVs (as participants with these SCVs present were excluded from the final sample and no information about T2D related SCVs were given) could contribute to the observed differences between the CHD and T2D interactions relating to self-efficacy. Thus, it seems that, for one, there could exist some uncharted and subtle (Cohen’s d = 0.14) phenomena between genome-wide polygenic risk and self-efficacy that do not manifest in the context of T2D risk. Alternatively, the effect could depend on knowledge of not having certain risk influencing SCVs. Lastly, it could be some combination of the two or a spurious finding. Consequently, we suggest that the current finding is first replicated in other studies before drawing more far-reaching conclusions about self-efficacy and genome-wide polygenic risk for CHD.

The second interaction also emerged only in the context of CHD risk. Here, worry related to traditional risk factors concerning CHD increased in both groups when low and elevated risk participants were compared and then slightly decreased when elevated and high risk participants were compared. Although the trend looked similar in both the control and experimental group, the upward spike from low to elevated risk was more pronounced in the group receiving genetic and traditional risk information. Moreover, pairwise comparisons indicated that the only significant differences were within the experimental group and not between the groups. Thus, it seems that there was more fluctuation between respondents’ reactions at different risk levels in the group receiving genetic and traditional risk information (producing significant within-group differences) but not enough to produce differences between the groups. That is, at any specific risk level, the control and experimental group did not formally differ from each other. Given that the observed pattern was only present in the context of CHD risk and not T2D risk, and no such effect emerged concerning worry related to genetic risk for either of the disease conditions, we suggest that the current finding is first replicated before drawing more firm conclusions.

While the type of risk information received did not produce consistent effects, the magnitude of risk (i.e., T2D/CHD risk level) did have a clear impact on nearly all the psychosocial variables studied. The effects generally flowed in the direction of what would be expected: on average, the higher risk was associated with higher perceived risk, worry, lower self-efficacy, and with less positive emotions and with more negative emotions. Effects concerning risk perception had the highest effect sizes (*η*p2 = 0.07–0.09), and extreme adverse reactions, such as respondents having difficulties enjoying life or feeling that they are losing control, had the smallest effect sizes (*η*p2 < 0.01). In general, adverse reactions to the risk estimates were subdued regardless of the risk level (e.g., the highest marginal mean observed concerning reactions with negative valence was M = 3.98 ± 0.19 for T2D related worry, which is just slightly above the middle point of the scale used).

It is possible that respondents with high or very high risk were already conscious of their heightened risk (e.g., due to lifestyle factors or family history), which might have made the emotional impact of the results less extreme. Likewise, since both conditions are actionable and the offered risk projection was for 10 years, respondents might have felt they still had time to make the necessary lifestyle changes and were thus not overly worried about their test results at present. Indeed, along with the risk estimates, participants were given information concerning lifestyle changes that could mitigate their disease risk. Importantly, the interactive calculator tangibly demonstrated how changing physical parameters and lifestyle factors could influence one’s risk for T2D and CHD. It is also conceivable that even when respondents had a high relative risk (i.e., risk contrasted with the population average), they might not have considered their absolute risk (e.g., 10–20%) to be high enough to cause great concern. Finally, it should be noted that only 34% of participants returned the pre-and post-survey on the same day. Thus, most respondents had some time to process the results, which might have softened the initial reaction.

To sum up, we did not find consistent evidence that distributing risk estimates based on a combination of genome-wide polygenic and traditional risk would cause psychological harm to the respondents. The presence of genome-wide polygenic risk did not seem to influence risk perception or cause an adverse emotional reaction. Likewise, self-efficacy and disease-related worry remained, for most parts, unaltered. The minute changes that did emerge (The highest ηp^2^ for significant effect involving the experimental and control group was 0.007, and the highest d was 0.14) could not be interpreted as harmful per se. As such, our results are largely in line with other studies concerning T2D (Grant et al., 2013; McVay et al., 2015; Voils et al., 2015; Godino et al., 2016) and CHD (Robinson et al., 2016) risk scores and with studies concerning polygenic risk scores more generally (Robinson et al., 2019; Wade, 2019). Results concerning the magnitude of the disease risk were consistent with expectations, albeit subdued.

Several limitations should be considered when interpreting the results as with any study. For one, absence of evidence is not evidence of absence (Altman and Bland, 1995). It is thus difficult to conclude with certainty that there are no harmful effects in receiving genome-wide polygenic risk information. However, given the sensitivity power analysis, our study does indicate that if these effects exist, they are likely relatively small in magnitude. Secondly, significant attrition occurred between the randomization and the post-survey (approximately 55% of respondents dropped), which exposed the sample to additional bias. Even though the observed differences in the measured socio-demographic variables were reasonably modest between those who dropped and stayed, the dropouts tended to be from groups that already suffer from underrepresentation in health surveys (e.g., less educated and with lower socioeconomic status; Reinikainen et al., 2018). Moreover, it is possible that the groups might also differ substantially from each other regarding some unmeasured factor. Thus, special caution should be applied when generalizing the results. Thirdly, as with most studies conducted in a real-life setting, the set-up included multiple potentially confounding factors. Notably, in the current study, respondents received multiple risk scores simultaneously (including SCV results), and response time was not strictly controlled (i.e., after receiving the notification, respondents themselves decided when to check their results and answer the survey questions). Although the sensitivity analyses did not indicate notable confounding effects, caution should still be applied when generalizing the results into a different context. Finally, it is important to keep in mind that when the risk estimates were communicated, the respondents also received information about risk-mitigating lifestyle strategies. The absence of this information or a change in the feedback could influence the results.

## Data Availability

The datasets presented in this study can be found in online repositories. The names of the repository/repositories and accession number(s) can be found below: https://thl.fi/en/web/thl-biobank/for-researchers, THL Biobank.

## Footnotes

1
https://thl.fi/en/web/thlfi-en/research-and-development/research-and-projects/the-p5.fi-study-genetic-information-for-health-support?redirect = %2Fen%2Fweb%2Fthlfi-en%2Fresearch-and-expertwork%2Fprojects-and-programmes%2Fcurrent-research-and-projects.
