# Supplementary material for: Psychosocial Effects of Receiving Genome-Wide Polygenic Risk Information Concerning Type 2 Diabetes and Coronary Heart Disease: A Randomized Controlled Trial
Source: Front Genet. 2022 May 30;13:881349. doi: 10.3389/fgene.2022.881349 (PMC9189371; doi:10.3389/fgene.2022.881349)
Supplement: Supplementary file 1 [file DataSheet1.PDF]

Supplementary File S1

1 Attrition analysis

To access attrition, respondents who dropped (i.e., who were randomized to experimental/control group but did not return the post-results survey; n = 1809) and who returned the post-results survey (n = 1368) were compared with each other. The chi-square test of independence indicated that respondents who stayed and who dropped differed in terms of all the socio-demographic variables (ps < .03; see Table 1). However, the size of the association was relatively small in all cases (Cramer's V = 0.04-0.21). Noteworthy, respondents who dropped were somewhat older (V = 0.21), less educated (V = 0.20), more likely to be pensioners (V = 0.16), had lower income (V = 0.17), and had a higher risk for CHD (V = 0.19). See Figure 1-2 for a visual comparison of different socio-demographic variables

Table 1. Comparison of different socio-demographic variables between those who dropped (N = 1809) after randomization and those who returned the post-results survey (N = 1368)

Variable	Chi-square	df	*p*	Cramer's V
Gender	5.00	1	.03	0.04
Age	132.15	6	<.001	0.21
Education	122.63	2	<.001	0.20
Occupation	76.44	4	<.001	0.16
Income	88.46	4	<.001	0.17
Marital status	8.62	2	.01	0.05
Number of children	7.76	1	.005	0.05
Group	24.52	1	<.001	0.09
T2D risk	17.36	3	<.001	0.08
CHD risk	105.67	2	<.001	0.19

1.1 Supplementary Figure 1

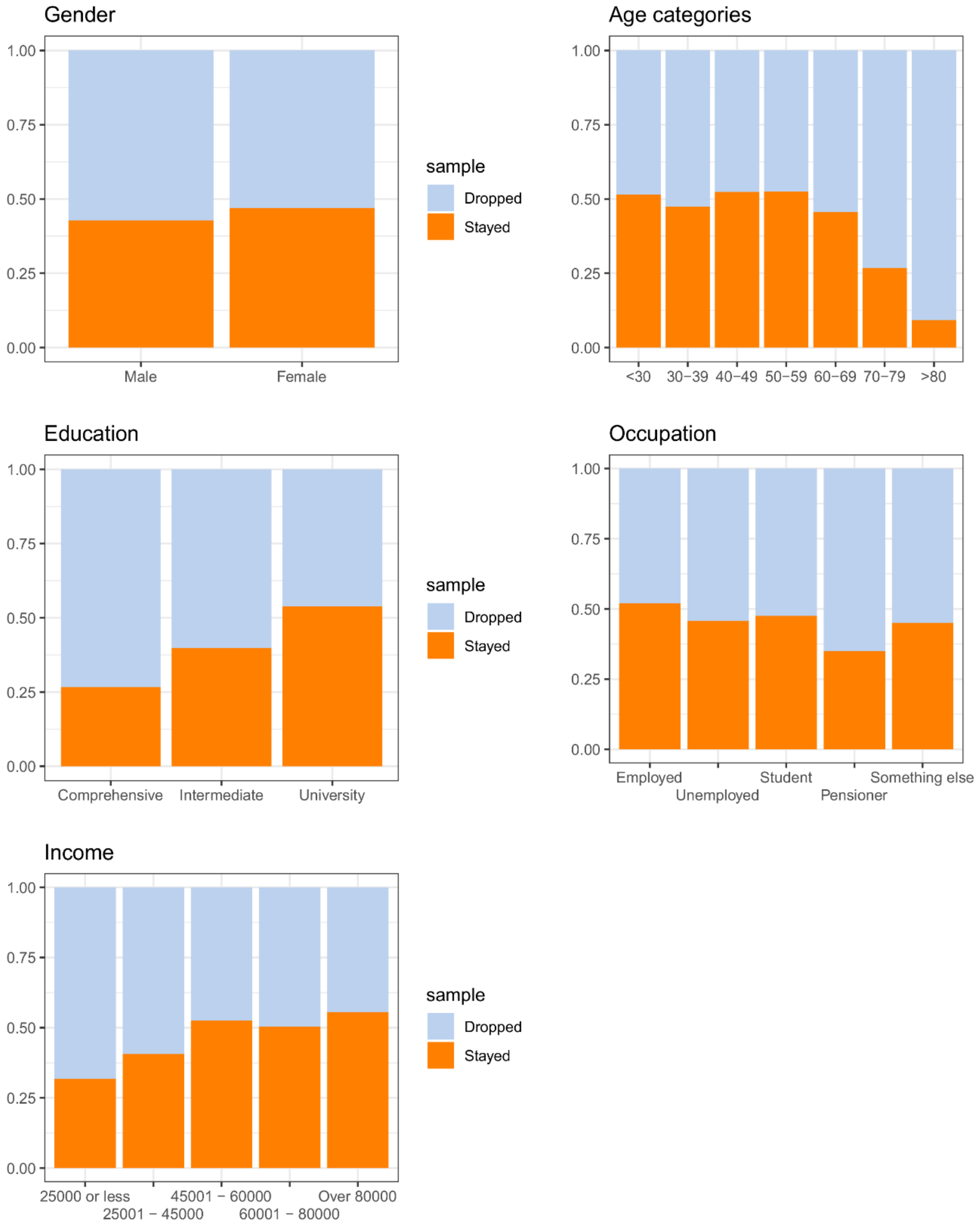

Supplementary Figure 1. Relative frequencies of the socio-demographic variables plotted for respondents who dropped after randomization and for those returned the post-results survey.

1.2 Supplementary Figure 2.

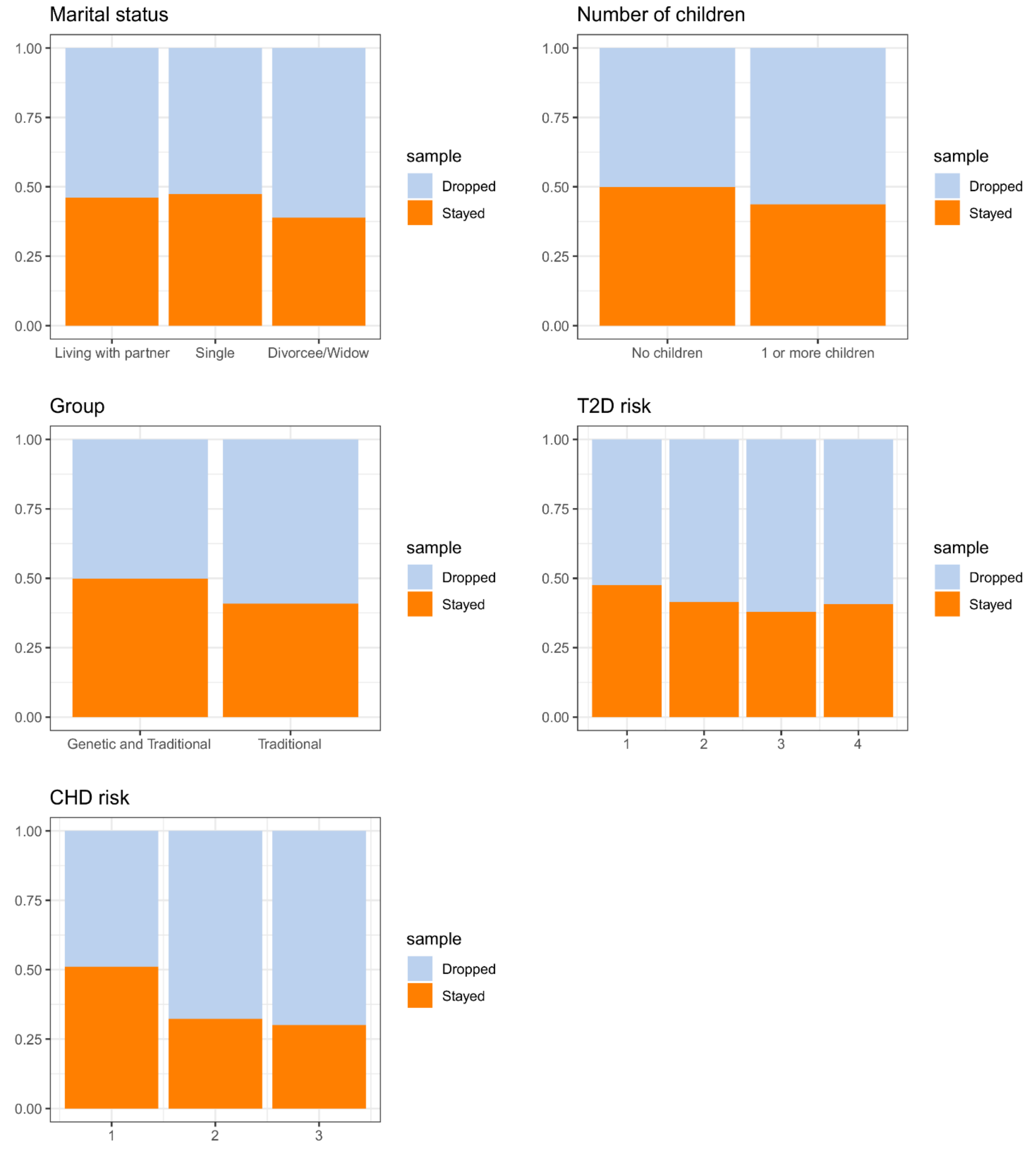

Supplementary Figure 2. Relative frequencies of the socio-demographic variables plotted for respondents who dropped after randomization and for those returned the post-results survey.

2 Sensitivity analysis: response time between pre- and post-results survey as a covariate

Since the response time (i.e., the time interval between the return of the pre- and the post-results survey) fluctuated between respondents and was not evenly distributed in the experiment and the control group (see Figure 3), it was decided to run a sensitivity analysis where the response time was added as a covariate in the models.

2.1 Supplementry Figure 3.

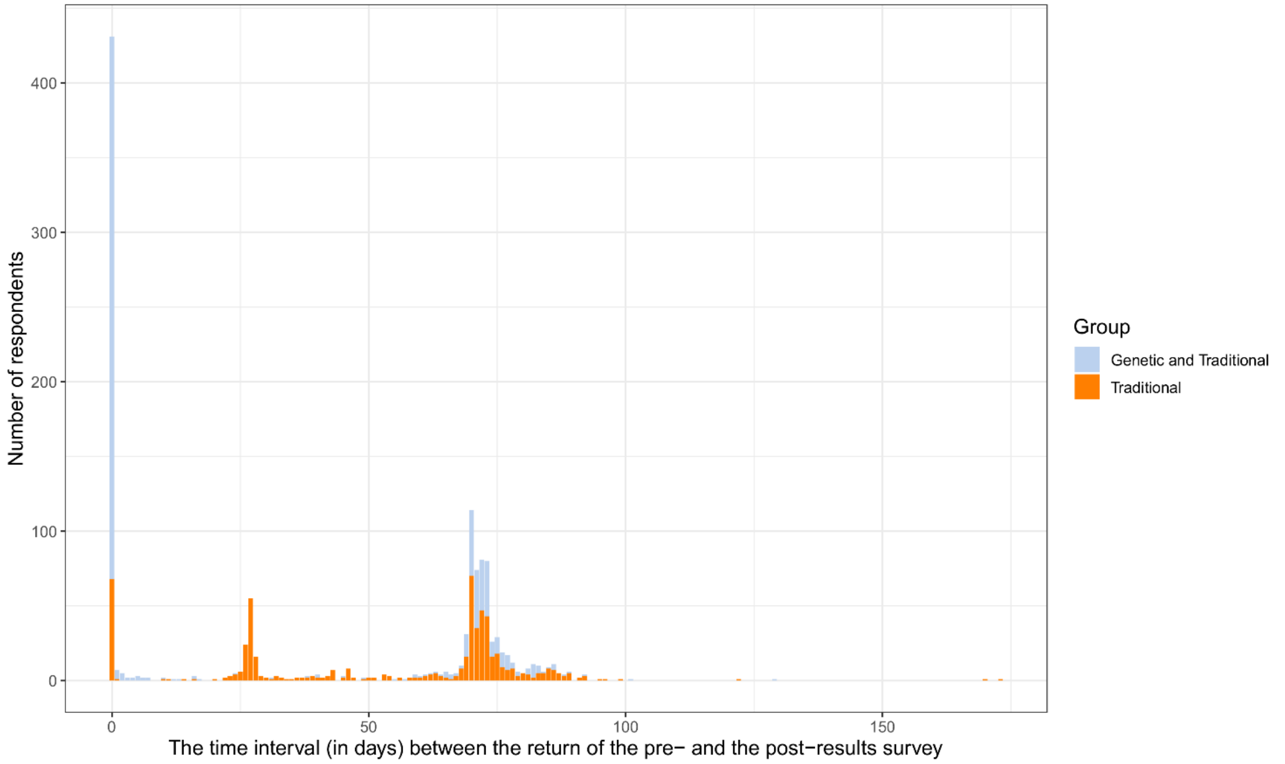

Supplementary Figure 3. Response time in the experiment and the control group.

As can be seen from Table 2, response time had a significant effect in only 2 of the 12 models tested (*ps* = .04, η_p^2 = 0.004). Moreover, in most models, the effect size for response time was only marginal (η_p^2 = 0.001 or less). Perhaps not surprisingly then, only minute changes occurred in the models after the addition of the response time. Risk types main effect remained nonsignificant in all models (*ps* > .11). Likewise, the interaction between risk type and CHD risk level concerning self-efficacy stayed statistically significant, and the same pattern of results was observed in estimated marginal means as before (see Figure 4). However, a small change was observed in relation to the interaction between the risk type and CHD risk level concerning worry related to traditional risk factors. After the addition of the response time, the p-value climbed just above the .05 alpha level (p = .06) and became statistically nonsignificant. Still, as shown in Figure 4, the pattern remained the same. We suspect that the change in the p-value (from .03 to .06) mainly reflects the fact that the sensitivity model had 64 fewer respondents due to missing values in the response time variable (on account of technical issues, some participants' response time was not accurately saved). The risk

level's main effect mainly stayed the same (see Figures 5-8). The only exceptions were that the CHD risk level did not anymore have a significant effect on how relieved the participants were (p = .053) and T2D risk level how much they felt loss of control after seeing the results (p = .09). We suspect that these changes are also mainly due to the differences in the sample size.

Table 2. Response time as a covariate: ANCOVA effects

		T2D				CHD			
**Dependent**	**Effect**	**F**	**df**	***p***	**η_p^2**	**F**	**df**	***p***	**η_p^2**
**Perceived risk**	Risk type	1.31	1, 1180	.25	0.001	0.20	1, 1218	.66	<0.001
	Risk level	32.83	3, 1180	<.001	0.077	55.94	2, 1218	<.001	0.084
	Risk type × Risk level	2.14	3, 1180	.09	0.005	0.08	2, 1218	.92	<0.001
	Covariate (pre)	659.36	1, 1180	<.001	0.358	603.10	1, 1218	<.001	0.331
	Covariate (time)	<0.01	1, 1180	.99	<0.001	4.47	1, 1218	.04	0.004
**Self-efficacy**	Risk type	0.04	1, 1184	.84	<0.001	0.53	1, 1199	.47	<0.001
	Risk level	2.09	3, 1184	.10	0.005	6.19	2, 1199	.002	0.010
	Risk type × Risk level	1.68	3, 1184	.17	0.004	4.03	2, 1199	.02	0.007
	Covariate (pre)	381.21	1, 1184	<.001	0.244	271.08	1, 1199	<.001	0.184
	Covariate (time)	1.12	1, 1184	.29	0.001	0.10	1, 1199	.75	<0.001
**Worry related to traditional risk**	Risk type	0.12	1, 1187	.73	<0.001	2.51	1, 1214	.11	0.002
	Risk level	29.49	3, 1187	<.001	0.069	19.44	2, 1214	<.001	0.031
	Risk type × Risk level	0.42	3, 1187	.74	0.001	2.85	2, 1214	.06	0.005
	Covariate (pre)	349.32	1, 1187	<.001	0.227	357.65	1, 1214	<.001	0.228
	Covariate (time)	0.25	1, 1187	.62	<0.001	0.26	1, 1214	.61	<0.001
**Worry related to genetic risk**	Risk type	0.14	1, 1169	.70	<0.001	0.06	1, 1198	.80	<0.001
	Risk level	20.63	3, 1169	<.001	0.050	21.93	2, 1198	<.001	0.035
	Risk type × Risk level	1.17	3, 1169	.32	0.003	1.23	2, 1198	.29	0.002
	Covariate (pre)	279.39	1, 1169	<.001	0.193	316.11	1, 1198	<.001	0.209
	Covariate (time)	2.07	1, 1169	.15	0.002	2.34	1, 1198	.13	0.002
**Upset**	Risk type	0.96	1, 1157	.33	0.001	0.73	1, 1178	.39	0.001
	Risk level	24.89	3, 1157	<.001	0.061	25.68	2, 1178	<.001	0.042
	Risk type × Risk level	0.31	3, 1157	.82	0.001	1.70	2, 1178	.18	0.003
	Covariate (time)	0.17	1, 1157	.68	<0.001	0.09	1, 1178	.76	<0.001
**Sad**	Risk type	0.52	1, 1155	.47	<0.001	0.07	1, 1176	.80	<0.001
	Risk level	12.28	3, 1155	<.001	0.031	18.10	2, 1176	<.001	0.030
	Risk type × Risk level	0.73	3, 1155	.53	0.002	1.09	2, 1176	.34	0.002
	Covariate (time)	0.23	1, 1155	.63	<0.001	0.10	1, 1176	.76	<0.001
**Nervous**	Risk type	0.98	1, 1153	.32	0.001	0.02	1, 1174	.89	<0.001
	Risk level	9.03	3, 1153	<.001	0.023	7.51	2, 1174	.001	0.013

	Risk type × Risk level	0.79	3, 1153	.50	0.002	0.67	2, 1174	.51	0.001
	Covariate (time)	0.16	1, 1153	.69	<0.001	0.28	1, 1174	.60	<0.001
**Guilt**	Risk type	0.07	1, 1156	.79	<0.001	1.23	1, 1176	.27	0.001
	Risk level	12.70	3, 1156	<.001	0.032	7.13	2, 1176	.001	0.012
	Risk type × Risk level	1.03	3, 1156	.38	0.003	0.27	2, 1176	.77	<0.001
	Covariate (time)	0.03	1, 1156	.87	<0.001	0.03	1, 1176	.87	<0.001
**Relieved**	Risk type	0.60	1, 1162	.44	0.001	<0.01	1, 1183	.99	<0.001
	Risk level	6.48	3, 1162	<.001	0.016	2.94	2, 1183	.05	0.005
	Risk type × Risk level	1.37	3, 1162	.25	0.004	0.04	2, 1183	.96	<0.001
	Covariate (time)	3.57	1, 1162	.06	0.003	2.91	1, 1183	.09	0.002
**Happy**	Risk type	0.01	1, 1157	.91	<0.001	0.42	1, 1178	.51	<0.001
	Risk level	16.65	3, 1157	<.001	0.041	14.93	2, 1178	<.001	0.025
	Risk type × Risk level	0.34	3, 1157	.80	0.001	0.07	2, 1178	.93	<0.001
	Covariate (time)	4.31	1, 1157	.04	0.004	3.01	1, 1178	.08	0.003
**Loss of control**	Risk type	0.56	1, 1156	.46	<0.001	0.43	1, 1176	.51	<0.001
	Risk level	2.17	3, 1156	.09	0.006	4.59	2, 1176	.01	0.008
	Risk type × Risk level	1.68	3, 1156	.17	0.004	0.80	2, 1176	.45	0.001
	Covariate (time)	0.08	1, 1156	.77	<0.001	0.05	1, 1176	.82	<0.001
**Difficulties enjoying life**	Risk type	1.19	1, 1152	.28	0.001	0.20	1, 1172	.66	<0.001
	Risk level	2.68	3, 1152	.046	0.007	3.53	2, 1172	.03	0.006
	Risk type × Risk level	0.61	3, 1152	.61	0.002	0.21	2, 1172	.81	<0.001
	Covariate (time)	0.76	1, 1152	.38	0.001	0.88	1, 1172	.35	0.001

Note. Covariate (time) refers to the time interval between the return of the pre- and the post-results survey measured in days. Covariate (pre) refers to the dependent variable measured in pre-results survey. Heteroskedasticity consistent covariate matrix HC3 is used in all models. P-values are Holm adjusted in each model. Partial eta squared is estimated based on degrees of freedom and F-values.

2.2 Supplementary Figure 4.

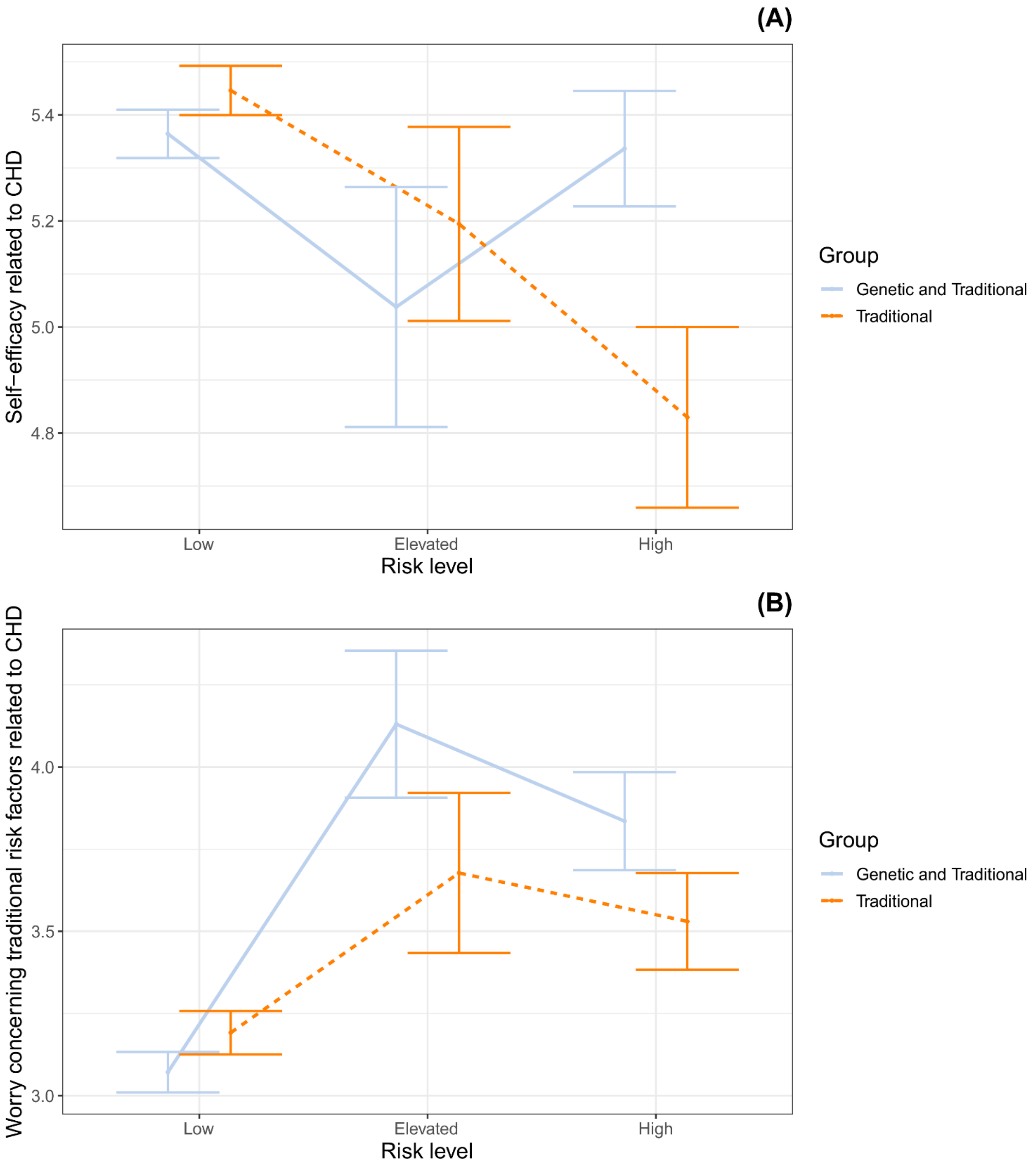

Supplementary Figure 4. Response time as a covariate: estimated means and standard errors for the interactions between risk type and CHD risk concerning self-efficacy (A) and worry related to traditional risk factors (B)

2.3 Supplementary Figure 5.

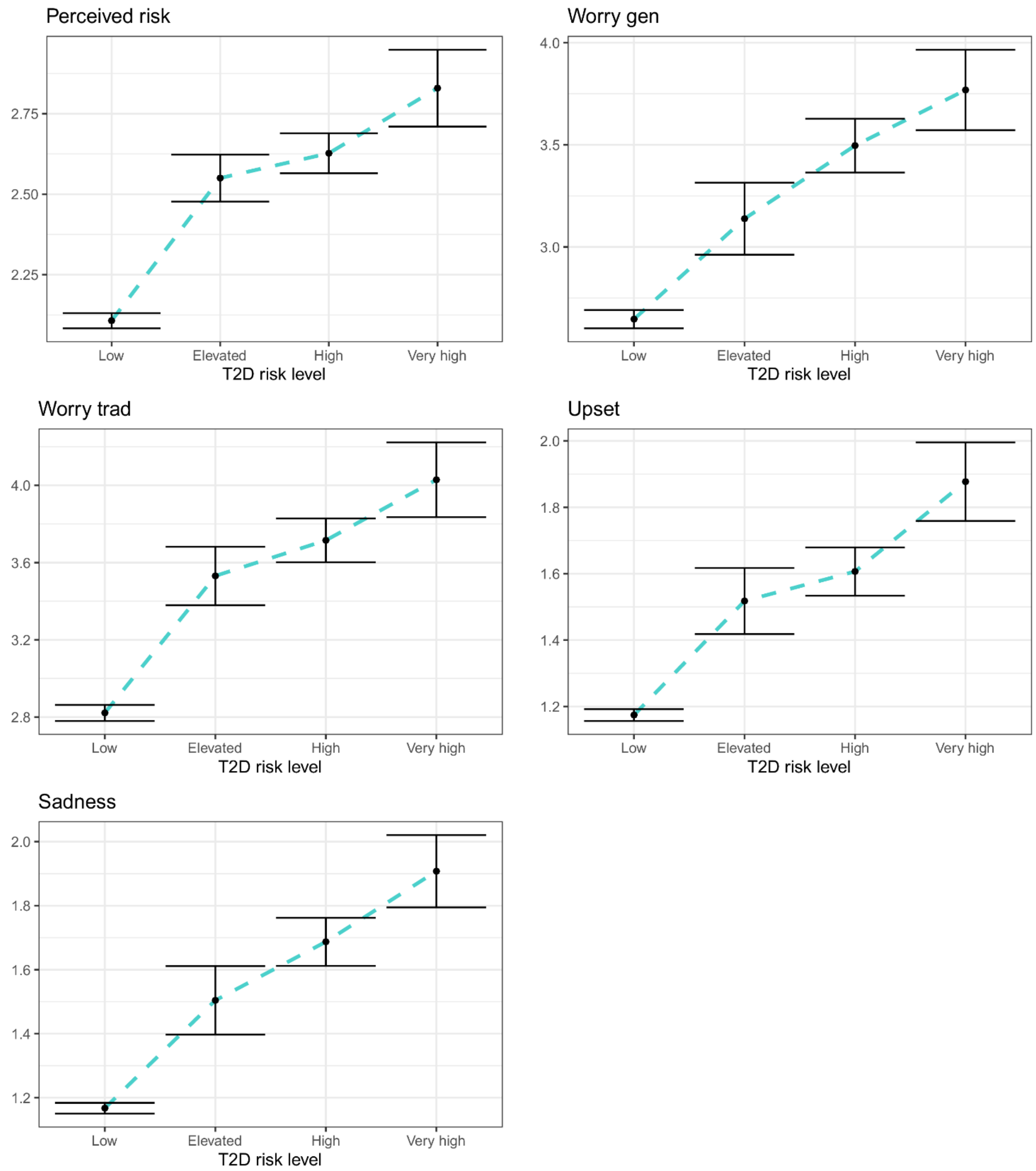

Supplementary Figure 5. Response time as a covariate: estimated means and standard errors for significant main effects of T2D risk (dependent variables: perceived risk, worry concerning traditional risk factors, worry concerning genetic risk factors, upset, sadness)

2.4 Supplementary Figure 6.

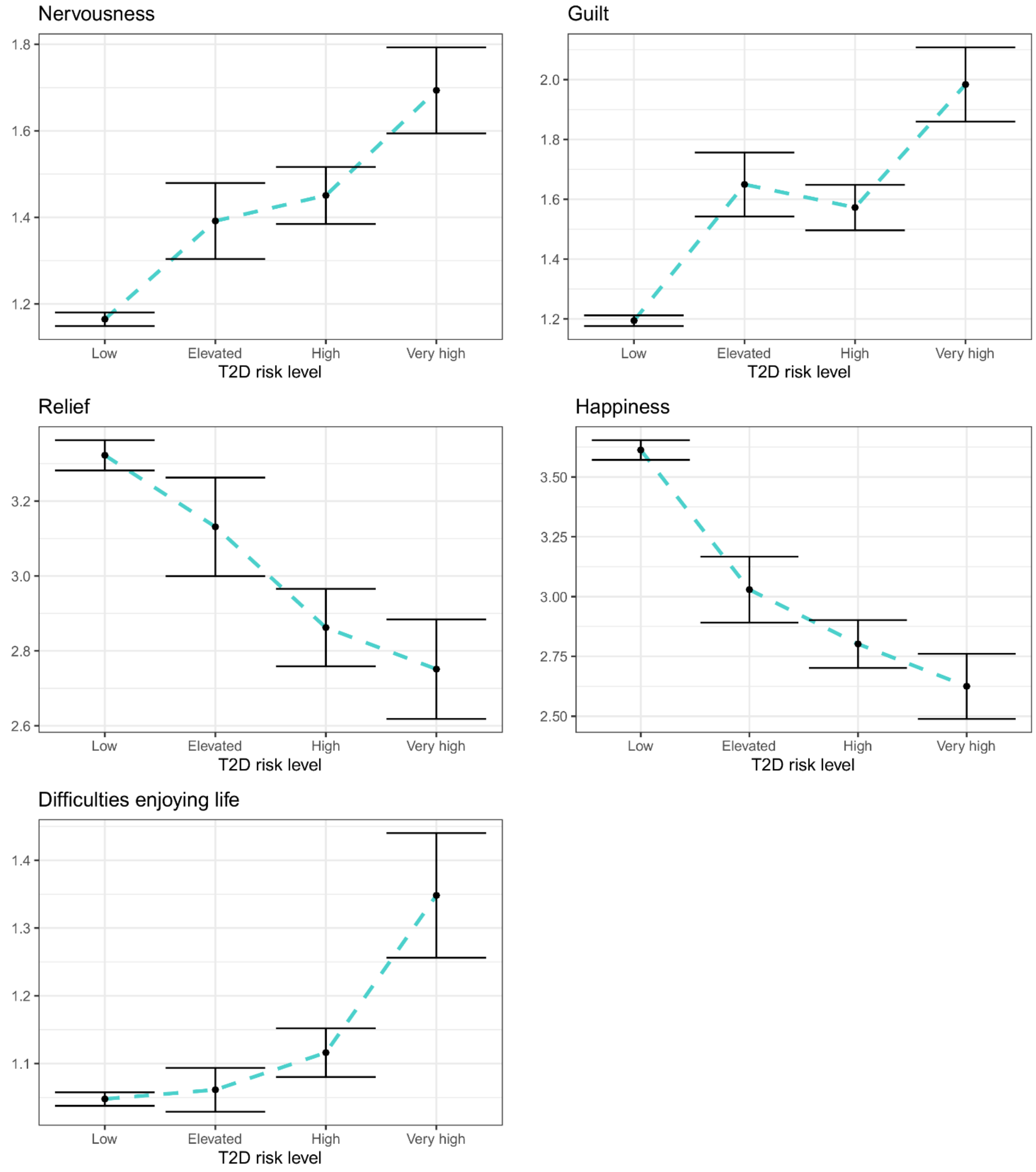

Supplementary Figure 6. Response time as a covariate: estimated means and standard errors for significant main effects of T2D risk (dependent variables: nervousness, guilt, relief, happiness, difficulties enjoying life)

2.5 Supplementary Figure 7.

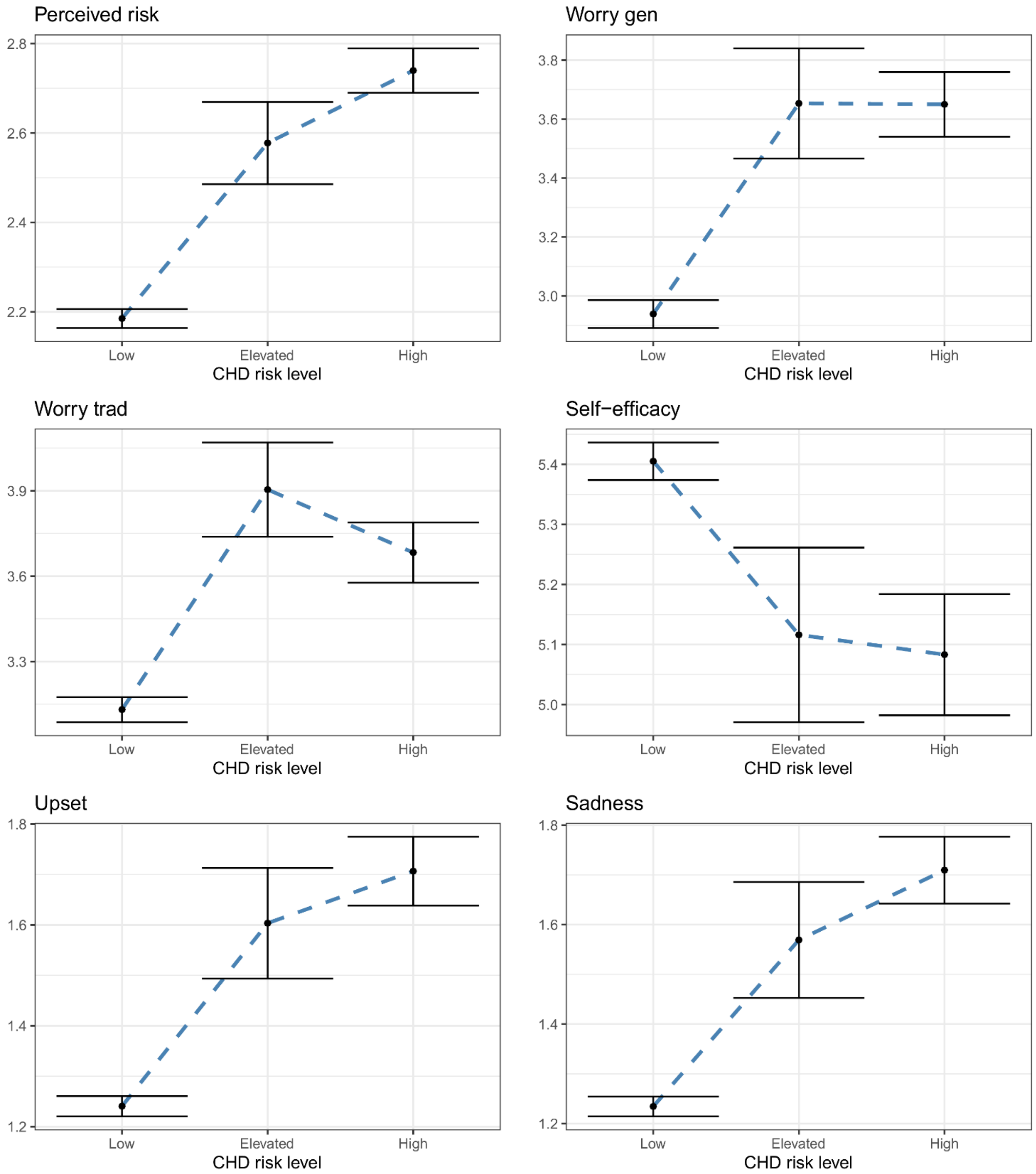

Supplementary Figure 7. Response time as a covariate: estimated means and standard errors for significant main effects of CHD risk (dependent variables: perceived risk, worry related to traditional factors, worry related to genetic factors, self-efficacy, upset, sadness)

2.6 Supplementary Figure 8.

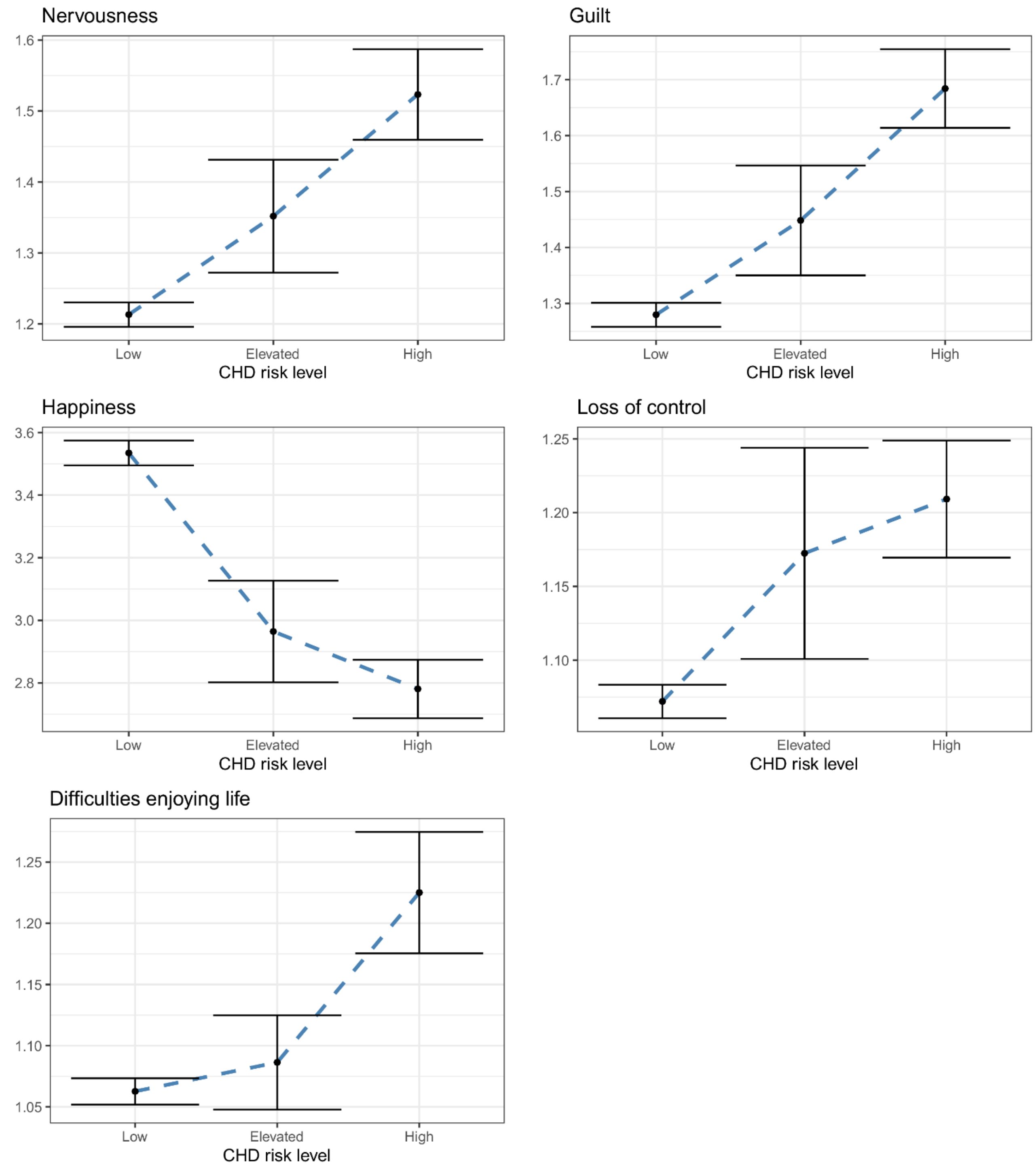

Supplementary Figure 8. Response time as a covariate: estimated means and standard errors for significant main effects of CHD risk (dependent variables: nervousness, guilt, happiness, loss of control, difficulties enjoying life)

3 Sensitivity analysis: Three-way ANOVA

The MICRA items were analyzed with all possible factors included (i.e., type of risk, T2D risk level, and CHD risk level). Importantly, no significant three-way interactions emerged (all *p*s > .34; see Table 3). Likewise, the type of risk had a nonsignificant main effect in all models (all *p*s > .39).

Table 3. Three-way ANOVA effects

Dependent	Effect	F	df	*p*	η_p^2	Dependent	Effect	F	df	*p*	η_p^2
	Risk type	0.73	1, 1167	.39	0.001		Risk type	0.03	1, 1165	.86	<0.001
	T2D level	4.19	3, 1167	.006	0.011		T2D level	1.17	3, 1165	.32	0.003
	CHD level	0.24	2, 1167	.78	<0.001		CHD level	2.01	2, 1165	.14	0.003
**Upset**	Risk type × T2D	0.10	3, 1167	.96	<0.001	**Sad**	Risk type × T2D	2.18	3, 1165	.09	0.006
	Risk type × CHD	1.27	2, 1167	.28	0.002		Risk type × CHD	1.27	2, 1165	.28	0.002
	T2D × CHD	0.80	6, 1167	.57	0.004		T2D × CHD	2.16	6, 1165	.04	0.011
	Rt × T2D × CHD	0.17	6, 1167	.99	0.001		Rt × T2D × CHD	0.67	6, 1165	.67	0.003
	Risk type	0.13	1, 1163	.72	<.001		Risk type	0.26	1, 1165	.61	<0.001
	T2D level	2.63	3, 1163	.049	0.007		T2D level	4.09	3, 1165	.007	0.010
	CHD level	1.59	2, 1163	.20	0.003		CHD level	1.33	2, 1165	.27	0.002
**Nervous**	Risk type × T2D	1.64	3, 1163	.18	0.004	**Guilt**	Risk type × T2D	0.95	3, 1165	.42	0.002
	Risk type × CHD	0.79	2, 1163	.46	0.001		Risk type × CHD	0.11	2, 1165	.89	<0.001
	T2D × CHD	2.43	6, 1163	.02	0.012		T2D × CHD	0.72	6, 1165	.64	0.004
	Rt × T2D × CHD	1.13	6, 1163	.34	0.006		Rt × T2D × CHD	0.35	6, 1165	.91	0.002
	Risk type	<0.01	1, 1171	.99	<0.001		Risk type	0.74	1, 1168	.39	0.001
	T2D level	1.36	3, 1171	.26	0.003		T2D level	1.62	3, 1168	.18	0.004
	CHD level	0.18	2, 1171	.83	<0.001		CHD level	0.99	2, 1168	.37	0.002
**Relieved**	Risk type × T2D	0.71	3, 1171	.54	0.002	**Happy**	Risk type × T2D	0.16	3, 1168	.92	<0.001
	Risk type × CHD	0.31	2, 1171	.73	0.001		Risk type × CHD	0.49	2, 1168	.61	0.001
	T2D × CHD	2.29	6, 1171	.03	0.012		T2D × CHD	1.91	6, 1168	.08	0.010
	Rt × T2D × CHD	0.41	6, 1171	.87	0.002		Rt × T2D × CHD	0.32	6, 1168	.93	0.002
	Risk type	0.11	1, 1164	.74	<0.001		Risk type	0.30	1, 1160	.58	<0.001
	T2D level	0.98	3, 1164	.40	0.003		T2D level	1.14	3, 1160	.33	0.003
	CHD level	2.78	2, 1164	.06	0.005		CHD level	2.11	2, 1160	.12	0.004
**Loss of control**	Risk type × T2D	1.76	3, 1164	.15	0.005	**Difficulties enjoying life**	Risk type × T2D	0.49	3, 1160	.69	0.001
	Risk type × CHD	0.92	2, 1164	.40	0.002		Risk type × CHD	0.12	2, 1160	.89	<0.001
	T2D × CHD	0.63	6, 1164	.71	0.003		T2D × CHD	1.43	6, 1160	.20	0.007
	Rt × T2D × CHD	1.10	6, 1164	.36	0.006		Rt × T2D × CHD	0.50	6, 1160	.81	0.003

Note. Heteroskedasticity consistent covariate matrix HC3 is used in all models. P-values are Holm adjusted in each model. Partial eta squared is estimated based on degrees of freedom and F-values.

However, a notable difference compared to the two-way models did emerge concerning risk level's influence. When T2D and CHD risk levels were in the same model, CHD risk did not have a significant main effect or interaction with risk type in any MICRA models (all *ps* > .06). T2D risk level's main effect remained significant concerning how upset, nervous, or guilty the participants felt (*p*s <. 05; other *ps* > .18; see Figure 9). No significant T2D risk level and risk type interactions emerged (*ps* > .09). Finally, when the interaction between T2D and CHD risk levels was inspected, significant interaction emerged concerning how sad, nervous, or relieved the participants felt (*ps* < .04; see Figure 10).

3.1 Supplementary Figure 9.

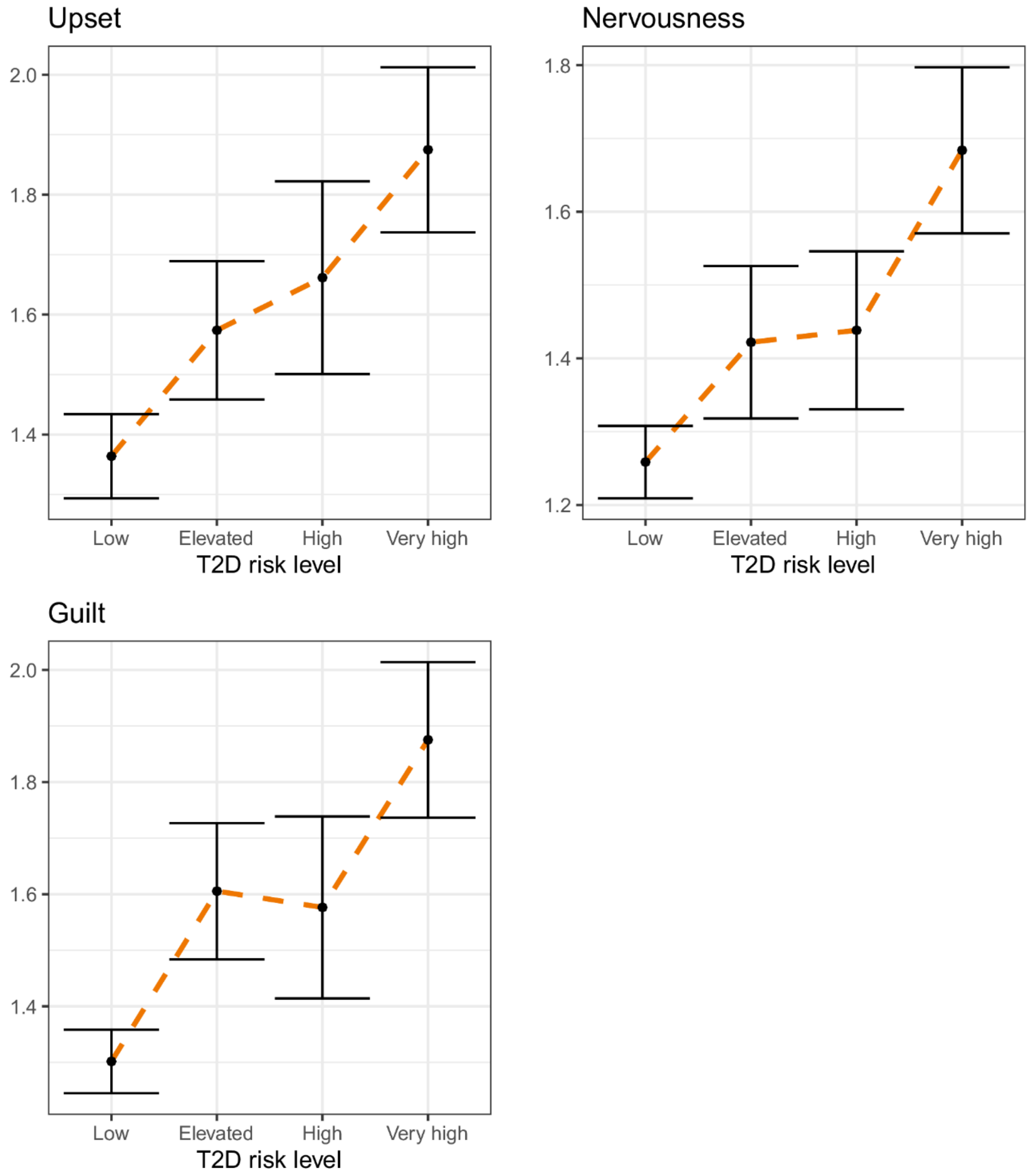

Supplementary Figure 9. 3-way ANOVA: estimated means and standard errors for significant main effects of risk level

3.2 Supplementary Figure 10.

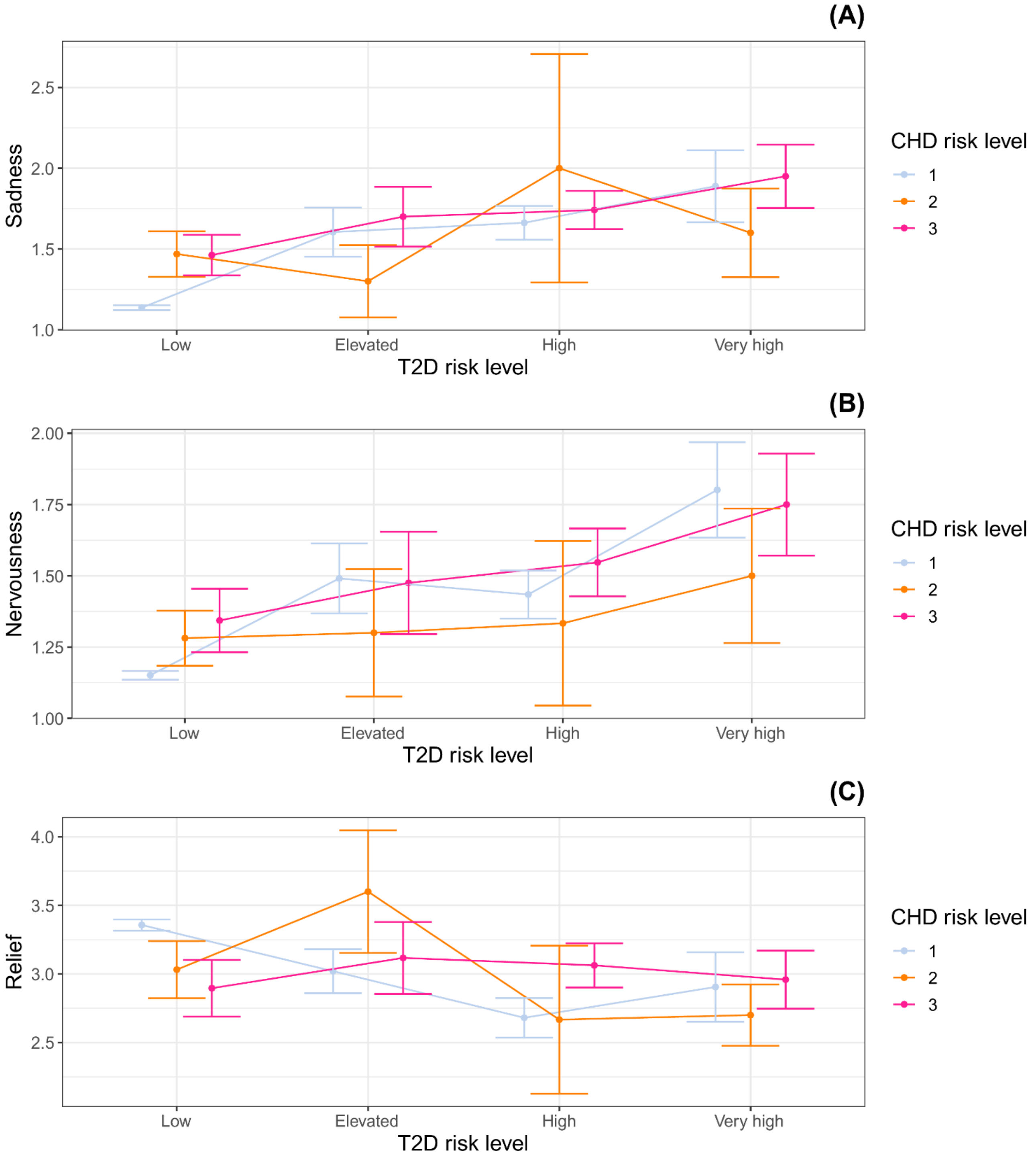

Supplementary Figure 10. 3-way ANOVA: estimated means and standard errors for significant T2D x CHD interaction effects
